# Genomic data provides insights into the evolutionary history and adaptive differentiation of two tetraploid strawberries

**DOI:** 10.1093/hr/uhae194

**Published:** 2024-07-11

**Authors:** Hanyang Lin, Luxi Chen, Chaonan Cai, Junxia Ma, Junmin Li, Tia-Lynn Ashman, Aaron Liston, Ming Dong

**Affiliations:** School of Advanced Study, Taizhou University, Taizhou 318000, China; Zhejiang Provincial Key Laboratory of Plant Evolutionary Ecology and Conservation, School of Life Sciences, Taizhou University, Taizhou 318000, China; Zhejiang Provincial Key Laboratory of Plant Evolutionary Ecology and Conservation, School of Life Sciences, Taizhou University, Taizhou 318000, China; Zhejiang Provincial Key Laboratory of Plant Evolutionary Ecology and Conservation, School of Life Sciences, Taizhou University, Taizhou 318000, China; Zhejiang Provincial Key Laboratory of Plant Evolutionary Ecology and Conservation, School of Life Sciences, Taizhou University, Taizhou 318000, China; School of Advanced Study, Taizhou University, Taizhou 318000, China; Zhejiang Provincial Key Laboratory of Plant Evolutionary Ecology and Conservation, School of Life Sciences, Taizhou University, Taizhou 318000, China; Department of Biological Sciences, University of Pittsburgh, Pittsburgh, PA 15260, USA; Department of Botany and Plant Pathology, Oregon State University, Corvallis, OR 97331, USA; Key Laboratory of Hangzhou City for Ecosystem Protection and Restoration, College of Life and Environmental Sciences, Hangzhou Normal University, Hangzhou 311121, China

## Abstract

Over the decades, evolutionists and ecologists have shown intense interest in the role of polyploidization in plant evolution. Without clear knowledge of the diploid ancestor(s) of polyploids, we would not be able to answer fundamental ecological questions such as the evolution of niche differences between them or its underlying genetic basis. Here, we explored the evolutionary history of two *Fragaria* tetraploids, *Fragaria corymbosa* and *Fragaria moupinensis*. We *de novo* assembled five genomes including these two tetraploids and three diploid relatives. Based on multiple lines of evidence, we found no evidence of subgenomes in either of the two tetraploids, suggesting autopolyploid origins. We determined that *Fragaria chinensis* was the diploid ancestor of *F. corymbosa* while either an extinct species affinitive to *F. chinensis* or an unsampled population of *F. chinensis* could be the progenitor of *F. moupinensis*. Meanwhile, we found introgression signals between *F. chinensis* and *Fragaria pentaphylla*, leading to the genomic similarity between these two diploids. Compared to *F. chinensis*, gene families related to high ultraviolet (UV)-B and DNA repair were expanded, while those that responded towards abiotic and biotic stresses (such as salt stress, wounding, and various pathogens) were contracted in both tetraploids. Furthermore, the two tetraploids tended to down-regulate defense response genes but up-regulate UV-B response, DNA repairing, and cell division gene expression compared to *F. chinensis*. These findings may reflect adaptions toward high-altitude habitats. In summary, our work provides insights into the genome evolution of wild *Fragaria* tetraploids and opens up an avenue for future works to answer deeper evolutionary and ecological questions regarding the strawberry genus.

## Introduction

Polyploidization (also known as whole-genome duplication) refers to the possession of more than two copies of each chromosome [1]. Polyploidization is widespread across eukaryotes, especially in plants [2, 3]. For instance, all extant species of vascular plants have experienced two ancient polyploidization events (ζ and ε), while monocots and dicots have undergone the other two (τ and γ, respectively) after their divergence [4]. Therefore, polyploidization is acknowledged to be one of the key factors promoting the evolution of higher plant genomes, the generation of new species, and the emergence of adaptive evolution [2, 5, 6].

Historically, some researchers have argued that polyploidization may lead to ‘evolutionary dead-ends’ [7]. Shortly after the polyploidization events, polyploids would exhibit lower rates of speciation and higher rates of extinction compared to their diploid relatives, further losing evolutionary potential [8]. However, mounting evidence has shown that polyploid species show stronger evolutionary advantages in the long term [6, 9]. Compared to their diploid relatives, polyploids tend to show stronger adaptability and a broader ecological niche, especially with respect to harsh environments (such as drought and extreme temperature) [10–13]. Nevertheless, due to the biased research interests (mainly cultivated plants, e.g., cotton [14]; sugarcane [15]; banana [16]) and the lack of sufficient genomic data, diploid ancestors of many wild polyploids await discovery. In addition, whether and how polyploids show differential responses to extreme habitats compared to their diploid relatives remains an open but intriguing question.

Harboring about 25 species with diverse ploidy levels (ranging from diploid [2×], to tetraploid [4×] and above [5×, 6×, 8×, 10×]), strawberries (*Fragaria* L., Rosaceae) are not only important food plants but also serve as a promising system to study the above-mentioned issues [17, 18]. To date, the diploid ancestors of the octoploid cultivated strawberry (*Fragaria* × *ananassa*) have been intensively studied [19–26]. Lines of evidence indicate that one *F.* × *ananassa* subgenome is most closely related to the extant *Fragaria vesca*, one subgenome resembles *Fragaria iinumae* mostly, while the other two distinct subgenomes could be derived from extinct or unsampled species that are more affinitive to *F. iinumae* than *F. vesca.* Nevertheless, none of these works shed light on the niche evolution between polyploids and their potential diploid ancestors, leaving an evident knowledge gap that calls for deeper investigations. Meanwhile, much less attention has been paid to the evolutionary trajectory of wild tetraploid *Fragaria* species (such as *Fragaria corymbosa* and *Fragaria moupinensis*), and hence, their diploid ancestors remain ambiguous. The most comprehensive work was reported by Kamneva *et al.* [27]. Based on 257 low-copy nuclear markers of 20 diploid and polyploid *Fragaria* species, they used phylogenetic approaches to infer the evolutionary origins of polyploid strawberries. This study provided some evidence of the diploid ancestors of tetraploids, yet the robustness of the conclusion is relatively weak (the support probability did not exceed 50%), possibly owing to the widespread reticulate evolution in *Fragaria* (as shown by later works [28]) and the limitations of datasets. Despite the imperfections, the work of Kamneva *et al.* [27] provides us with valuable clues that *Fragaria chinensis* and *Fragaria pentaphylla* would be the likely ancestors of both *F. corymbosa* and *F. moupinensis.* Besides molecular analyses, morphological comparisons suggested that *F. moupinensis* most resembles *Fragaria nubicola*, and *F. chinensis* could be the ancestor of *F. corymbosa* [17, 29]. On the other hand, information on geographical distribution and earlier studies have suggested that these two Chinese endemic tetraploids (*F. corymbosa* and *F. moupinensis*) may have undergone climate niche evolution after the divergence from their diploid ancestors [30]. In general, *F. corymbosa* and *F. moupinensis* were prone to occupy higher altitude habitats with lower temperatures, compared to their diploid relatives [31]. Again, no solid conclusion should be drawn without a clear species phylogeny, and stronger genomic data are needed to decipher the evolutionary histories of the tetraploids and examine how their gene expression differs from the diploids in a manner that would facilitate niche differentiation.

**Table 1:** Plant information and genome characteristics of five sequenced *Fragaria* species.

Species	Collection locality	Latitude	Longitude	Evaluated genome size (Mb)	Assembly size (Mb)	Percentage of anchoring (%)	Contig N50 (Kb)	Scaffold N50 (Mb)	Repeats (%)	Number of predicted coding genes	Complete BUSCO (%)
*Fragaria corymbosa*	Gansu, China	35.776	103.964	236.99 (1×)	875.99 (4×)	98.32	408.52	31.54	44.41	81 282	98.0
*Fragaria moupinensis*	Sichuan, China	30.043	101.827	236.20 (1×)	980.12 (4×)	96.76	323.22	32.88	43.18	89 611	98.2
*Fragaria chinensis*	Shaanxi, China	33.279	108.302	236.27 (1×)	254.62 (1×)	92.54	2290.75	32.42	44.95	30 300	95.4
*Fragaria pentaphylla*	Sichuan, China	31.717	103.883	259.21 (1×)	253.27 (1×)	97.01	2365.18	34.20	43.56	33 501	96.2
*Fragaria daltoniana*	Xizang, China	28.029	85.986	273.67 (1×)	270.50 (1×)	96.69	6955.13	34.83	45.91	31 385	98.6

In the present study, we first used genomic data to determine the most likely diploid ancestors of *F. corymbosa* and *F. moupinensis*. Then, we tested whether and to what extent hybridization played a role in shaping the evolutionary history of the two tetraploids and their close relatives. Last, we investigated genes that show contrasting responses to the presumed climate niche divergence between pairs of ancestor–descendant species. By utilizing phylogenomic approaches, the current study sheds light on the evolutionary imprints of these wild *Fragaria* tetraploids with ecological significance.

## Results

### The characteristics of five assembled *Fragaria* genomes

**Figure 1:**
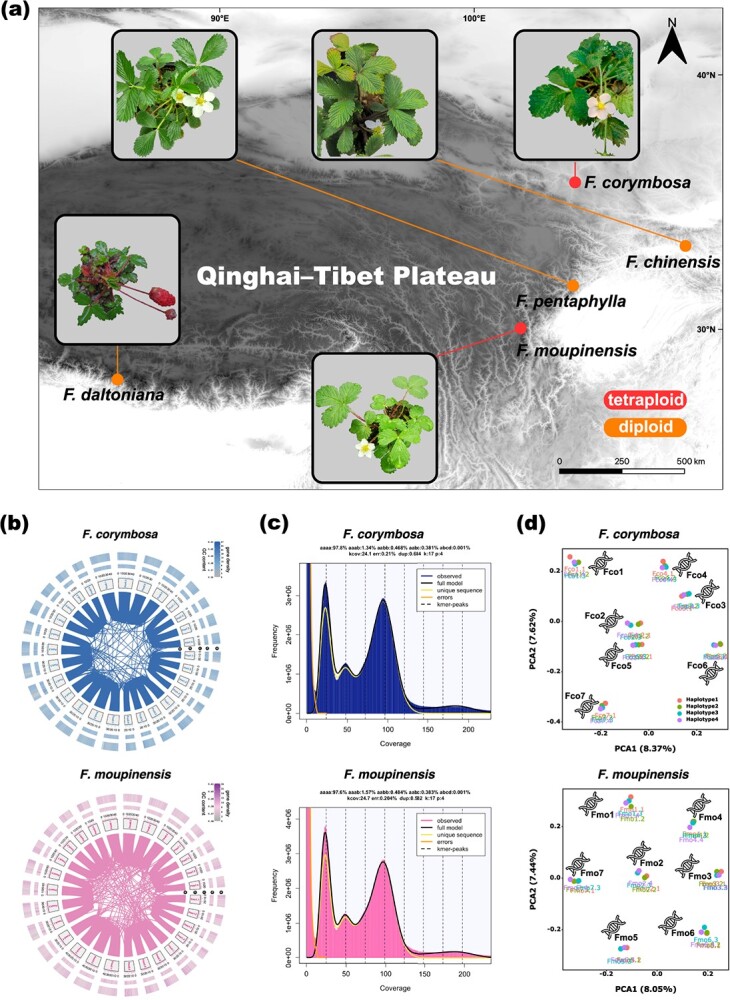
An overview of five sequenced *Fragaria* species. **(a)** The sampling localities and photographs of sequenced *Fragaria* individuals showing the overall morphological features and reproductive organs*.* Photo credits: the authors. **(b)** The genome features of *Fragaria corymbosa* and *Fragaria moupinensis* showing (i) gene synteny blocks; (ii) the labels of pseudo-chromosomes; (iii) sequence positions; (iv) GC contents; and (v) gene densities. **(c)** The *k*-mer spectra and fitted models of *F. corymbosa* and *F. moupinensis* genomes. **(d)** The distribution of TE profiles along the first and second axes of principal components.

The chromosome squashes confirmed the tetraploidy of both sequenced *F. corymbosa* and *F. moupinensis* individuals (Fig. S1, see online supplementary material). Through the *de novo* assembly pipeline, we achieved initial genome assemblies that captured 875.99, 980.12, 254.62, 253.27, and 270.50 Mb in 2956, 6692, 382, 221, and 185 contigs for *F. corymbosa*, *F. moupinensis*, *F. chinensis*, *F. pentaphylla*, and *Fragaria daltoniana* genomes, with contig N50 of 408.52, 323.22, 2290.75, 2365.18, and 6955.13 Kb, respectively (Table 1). The assembled genome sizes are largely consistent with estimated values from the genome survey. Using the Hi-C assisted scaffolding pipeline, 861.25 Mb which accounted for 98.32% of the assembled *F. corymbosa* genome, 948.39 Mb which accounted for 96.76% of the assembled *F. moupinensis* genome, 235.62 Mb which accounted for 92.54% of the assembled *F. chinensis* genome, 245.70 Mb which accounted for 97.01% of the assembled *F. pentaphylla* genome, and 261.56 Mb which accounted for 96.69% of the assembled *F. daltoniana* genome were anchored on 28 (for the two tetraploids) or seven (for the three diploids) pseudo-chromosomes (Table 1; Fig. S2, see online supplementary material). The BUSCO evaluation suggested that these genomes were well-assembled (complete BUSCOs >95%; Table 1). With the hybrid annotation procedure, we predicted 81 282, 89 611, 30 300, 33 501, and 31 385 coding genes in the *F. corymbosa* genome, the *F. moupinensis* genome, the *F. chinensis* genome, the *F. pentaphylla* genome, and the *F. daltoniana* genome, respectively (Table 1). The size of repeat sequences contributed to 44.41% of the *F. corymbosa* genome, 43.18% of the *F. moupinensis* genome, 44.95% of the *F. chinensis* genome, 43.56% of the *F. pentaphylla* genome, and 45.91% of the *F. daltoniana* genome. For the two tetraploids, we found no presence of subgenomes based on the gene synteny analysis, the *k*-mer spectrums, and the principal components analysis (PCA) results of the transposable elements (TE) profiles (Fig. 1). Specifically, we observed a higher proportion of *aaab* than *aabb* in both genomes of *F. corymbosa* (1.34% vs. 0.468%) and *F. moupinensis* (1.57% vs. 0.484%) and two major peaks in the *k*-mer distribution with 1/4 coverage ratio, indicating that these two species are autotetraploids.

**Figure 2:**
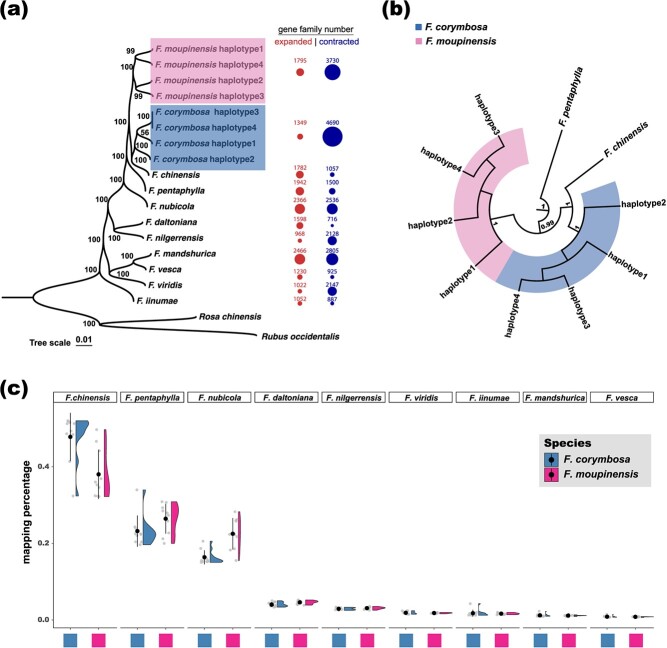
Identification of the most likely diploid ancestors of the two *Fragaria* tetraploids. **(a)** The consensus ML tree based on the concatenated sequences of 6054 single-copy orthologous genes. The bootstrap values are shown near nodes. The numbers of expanded (red) or contracted (blue) gene families of each *Fragaria* species are shown on the right of the tip labels. **(b)** The ASTRAL species tree regarding the two tetraploids and their close relatives summarized from 6054 ML gene trees. The values of local posterior probability are shown. **(c)** Reads mapping rates of *Fragaria corymbosa* and *Fragaria moupinensis* individuals to the composite genome constituted by nine *Fragaria* diploids shown by the sppIDer analysis.

### The diploid progenitor of two tetraploids

With the inclusion of 19 studied species, we obtained 27 (present in all 25 haplotypes), 520 (≥13 haplotypes), and 2587 (≥4 haplotypes) single-copy orthologous genes (OGs) based on different thresholds of haplotype coverages. Both the consensus ML tree based on the concatenated sequences and the ASTRAL species tree strongly suggested the monophyly of the *Fragaria* genus, and close evolutionary relationships among *Rosa chinensis*, *Rubus occidentalis*, and *Fragaria* species [Fig. S3, see online supplementary material; bootstrap (BS) value = 100, local posterior probability (LPP) = 1.00]. Subsequently, we identified 851 (present at all 19 haplotypes), 4045 (≥10 haplotypes), and 6054 (≥4 haplotypes) single-copy OGs among *Fragaria*, *R. chinensis*, and *R. occidentalis* with three haplotype coverages. The reconstructed phylogeny based on these three OG datasets and both the concatenation and the coalescent-based methods were highly congruent, and strongly supported the monophyly (BS value = 100, LPP = 1.00) of the clade comprising *F. corymbosa*, *F. chinensis*, *F. moupinensis*, *F. pentaphylla*, and *F. nubicola* (Fig. 2a and b; Fig. S4, see online supplementary material). Within this clade, *F. nubicola* represented the first diverged species; then *F. pentaphylla* diverged and *F. moupinensis* showed a sister relationship with the *F. chinensis*–*F. corymbosa* species pair (Fig. 2a and b; BS value = 100, LPP = 1.00). Also, all haplotypes within *F. corymbosa* and *F. moupinensis* showed strong monophyly (BS value = 100, LPP = 1.00), implying the absence of subgenomes.

The sppIDer analysis generated similar results with the ‘tree-based’ methods as shown above (Fig. 2c). Both the two tetraploids showed greater genomic similarity with *F. chinensis*, *F. pentaphylla*, and *F. nubicola* compared to other *Fragaria* diploids (Fig. 2c; Table S1, see online supplementary material). After mapping genomic sequences of *F. corymbosa* to the composite genome, we found that the largest proportion of the reads (with an average of 47.8%, hereafter) showed similarity with the *F. chinensis* genome (*df* = 8, *F* = 371.4, ANOVA *P*-value <0.001, Tukey’s HSD *P*-value <0.001), followed by *F. pentaphylla* (23.2%) and *F. nubicola* (16.4%). For *F. moupinensis*, we found that most of the reads (with an average of 38.0%) also mapped to the *F. chinensis* genome (*df* = 8, *F* = 1308, ANOVA *P*-value <0.001, Tukey’s HSD *P*-value <0.001), followed by *F. pentaphylla* (26.4%) and *F. nubicola* (22.5%). Therefore, we cautiously deduced that the ancestor–descendant relationship between *F. chinensis* and *F. corymbosa* was robust, and the most likely diploid ancestor of *F. moupinensis* could be an extinct species closely related to *F. chinensis* or an unsampled *F. chinensis* population.

### Introgression between *F. chinensis* and *F. pentaphylla*

**Figure 3:**
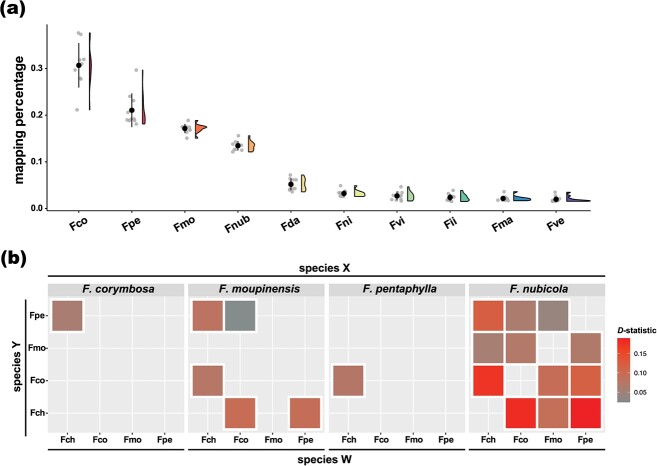
Genetic admixtures among the two tetraploids and their close relatives revealed by genome-resequencing data. **(a)** Reads mapping rates of *Fragaria chinensis* individuals to the composite genome constituted by ten *Fragaria* species shown by the sppIDer analysis. Fco = *Fragaria corymbosa*, Fpe = *Fragaria pentaphylla*, Fmo = *Fragaria moupinensis*, Fnu = *Fragaria nubicola*, Fda = *Fragaria daltoniana*, Fni = *Fragaria nilgerrensis*, Fvi = *Fragaria viridis*, Fii = *Fragaria iinumae*, Fma = *Fragaria mandshurica*, Fve = *Fragaria vesca*. **(b)** The *D*-statistics among these species. *F. nilgerrensis* was designated as the outgroup consistently. A warmer color indicates a greater *D*-statistic hence a stronger signal of introgression. Only significant *D*-statistics (*Z*-score > 3) are shown.

Using the ‘sequence-based’ method, we found that the genomic sequences of *F. chinensis* most resembled the *F. corymbosa* genome (30.7%; *df* = 9, *F* = 238.4, ANOVA *P*-value <0.001, Tukey’s HSD *P*-value <0.001) and followed by the *F. pentaphylla* genome (21.1%) (Fig. 3a; Table S1, see online supplementary material).

**Figure 4:**
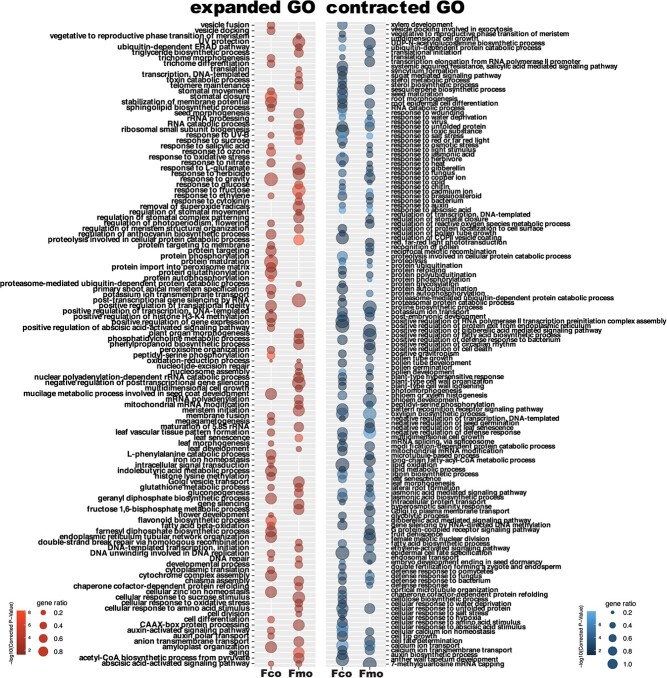
Enriched GO terms of genes that exclusively expanded (the left panel, red) or contracted (the right panel, blue) in *Fragaria corymbosa* (Fco) and *Fragaria moupinensis* (Fmo) compared to *Fragaria chinensis*. The heatmap corresponds to the *p*-value of Fisher’s exact test (with Benjamini and Hochberg FDR correction). The dot size corresponds to the gene ratio.

After the mapping and filtering processes, we retained 285 066 single-nucleotide polymorphism (SNP) loci to infer the *D*-statistics among focal species. With *F. corymbosa* being the species X, we found excess allele sharing between *F. chinensis* and *F. pentaphylla* (*D*-statistic = 0.0733, *Z*-score = 9.959), suggesting introgression between these two diploid species (Fig. 3b; Table S2, see online supplementary material). With *F. moupinensis* being the species X, we observed excess allele sharing among *F. chinensis*, *F. pentaphylla*, and *F. corymbosa*. With *F. pentaphylla* being the species X, a high magnitude of allele sharing was suggested between *F. chinensis* and *F. corymbosa*. With *F. nubicola* being the species X, signals of introgression were shown in all species pairs, which is intuitive as the results of phylogenic analysis have shown that *F. nubicola* was relatively phylogenetically distant from the remaining *Fragaria* species (Fig. 2a). In this case, the strongest introgression signal was found between *F. pentaphylla* and *F. chinensis* (*D*-statistic = 0.1889, *Z*-score = 24.128; Fig. 3b; Table S2, see online supplementary material). To summarize, multiple lines of evidence suggest the presence of genomic introgression between *F. chinensis* and *F. pentaphylla*.

### Gene families with expansion or contraction in the tetraploids

The *K* = 2 model was determined to be the best model of gene family evolutionary rates in CAFE analysis based on the final likelihood (Table S3, see online supplementary material). We found that 1349 gene families expanded in size while 4690 gene families contracted in size in the *F. corymbosa* genome (Fig. 2a). Among those, 1335 gene families exclusively expanded and 4374 gene families exclusively contracted in *F. corymbosa* compared to *F. chinensis* (Fig. S5, see online supplementary material). Meanwhile, 1795 gene families expanded in size while 3730 gene families contracted in size in the *F. moupinensis* genome, among which 1624 expanded gene families and 3479 contracted gene families were not found in *F. chinensis* (Fig. S5, see online supplementary material).

The gene enrichment analysis showed that 61 and 62 Gene Ontology (GO) terms were enriched in the expanded gene families of *F. corymbosa* and *F. moupinensis*, respectively (Fig. 4; Table S4, see online supplementary material). Among those, GO terms related to the response to high ultraviolet (UV)-B, DNA repair, the abscisic acid-activated signaling pathway, and the glutathione metabolic process were both enriched in *F. corymbosa* and *F. moupinensis*. On the other hand, 96 and 83 GO terms were enriched in the contracted gene families of *F. corymbosa* and *F. moupinensis*, respectively (Fig. 4; Table S4, see online supplementary material). We found that many of the shared GO terms were related to the responses to abiotic and biotic stresses, such as response to wounding, response to salt stress, response to heat, defense response to fungus, defense response to bacterium, and defense response to oomycetes (Fig. 4; Table S4, see online supplementary material). These shared GO terms between *F. corymbosa* and *F. moupinensis* enriched from both the expanded and the contracted gene families may be relevant to the high-altitude habitat of these two tetraploids compared to their closest diploid ancestor.

### Up- and down-regulated genes between the tetraploids and their closest diploid relative

We identified 920 differentially expressed genes (DEGs) between the transcripts of *F. corymbosa* and *F. chinensis*, among which 643 DEGs were up-regulated and 277 DEGs were down-regulated in *F. corymbosa* (Fig. S6, see online supplementary material). Meanwhile, 1327 DEGs were found between the transcripts of *F. moupinensis* and *F. chinensis*, among which 793 DEGs were up-regulated and 534 DEGs were down-regulated in *F. moupinensis* (Fig. S6, see online supplementary material).

**Figure 5:**
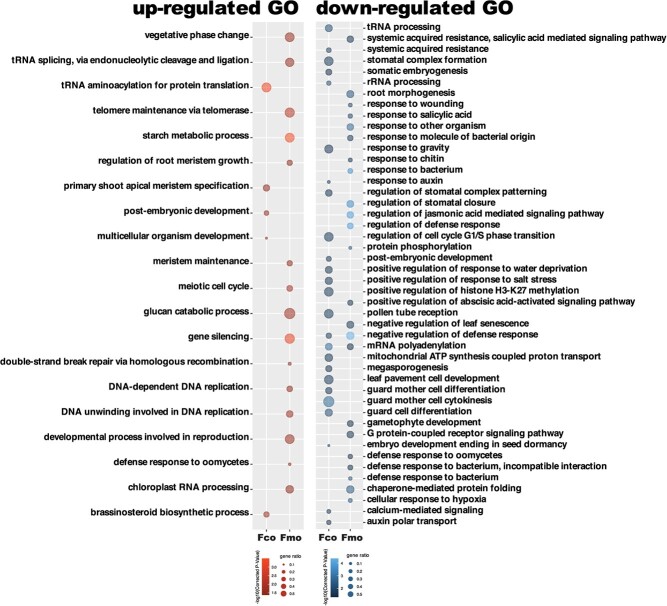
Enriched GO terms of genes that up-regulated (the left panel, red) or down-regulated (the right panel, blue) in *Fragaria corymbosa* (Fco) and *Fragaria moupinensis* (Fmo) compared to *Fragaria chinensis*. The heatmap corresponds to the *p*-value of Fisher’s exact test (with Benjamini and Hochberg FDR correction). The dot size corresponds to the gene ratio.

These up-regulated genes were enriched to 19 GO terms in total (Fig. 5; Table S5, see online supplementary material). Some GO terms were related to the processes of the cell division, such as telomere maintenance, meiotic cell cycle, DNA-templated DNA replication, and meristem maintenance, which may secure cell functions under UV-B damages. These down-regulated genes were enriched to 48 GO terms (Fig. 5; Table S5, see online supplementary material), many of which were related to the regulation of defense response and systemic acquired resistance. In general, the results of gene enrichment analysis of DEGs partly coincided with those of expanded or contracted genes (Figs 4 and 5).

## Discussion

By taking advantage of rapidly developing DNA sequencing technologies, we are now able to trace the evolutionary histories of these two tetraploids with whole-genome data. The reconstructed *Fragaria* phylogeny here is consistent with the work of Qiao *et al.* [32], which supported that *F. chinensis*, *F. pentaphylla*, and *F. nubicola* showed close evolutionary affinities and formed a monophyletic clade. With the inclusion of the two tetraploids, our phylogenomic analysis revealed a strongly supported clade that consists of *F. corymbosa*, *F. chinensis*, *F. moupinensis*, *F. pentaphylla*, and *F. nubicola* (Fig. 2a). Within this clade, *F. nubicola* and *F. pentaphylla* diverged sequentially; then the subclade was comprised of *F. moupinensis* and the *F. corymbosa*–*F. chinensis* pair. This result is also partly supported by Kamneva *et al.* [27] and morphological similarities among these species [17, 29]. Early studies have disputed whether *F. corymbosa* and *F. moupinensis* were autotetraploids or allotetraploids [33, 34]. In the paper of Kamneva *et al.* [27], they claimed that the hybridization events between *F. chinensis* and *F. pentaphylla* gave rise to the speciation of both *F. corymbosa* and *F. moupinensis*. However, this inference was weakly supported (the level of support was at most 20%). If the hypothesized scenario of Kamneva *et al.* [27] was true, then both the two tetraploids would at least show some signs of subgenomes that were supported by neither the present study (Figs 1 and 2) nor recent work [35].

By integrating lines of evidence from both the ‘tree-based’ method and the ‘sequence-based’ method [21], we deduce that *F. chinensis* is the sole diploid ancestor of *F. corymbosa* and is the closest diploid relative of *F. moupinensis*, though the genuine ancestor of *F. moupinensis* could be an extinct species affinitive to *F. chinensis* or an unsampled *F. chinensis* population (Fig. 2). The introgression between *F. chinensis* and *F. pentaphylla* could account for the reason why *F. chinensis* showed greater genomic similarities with *F. pentaphylla* than with *F. moupinensis* (Fig. 3). In the sense of biogeography, both *F. chinensis* and *F. pentaphylla* and their tetraploids relatives can be found in the flora surrounding the Qinghai-Tibet Plateau (QTP), where interspecific hybridization is prevailing (Fig. 1a; see Review in [36]). Therefore, the hybridization between *F. chinensis* and *F. pentaphylla* is feasible and has also been implicitly suggested by Feng *et al.* [28]. More recently, Qiao *et al.* [35] also found the presence of genetic admixtures between *F. chinensis* and *F. pentaphylla* and claimed the evolutionary affinity between *F. chinensis*, *F. pentaphylla*, and *F. corymbosa*. Here, we sequenced the genome of both tetraploids along with the comparisons between the expression levels of genes, which generates additional insights into the ancestor–descendant relationship of diploid-tetraploid species.

The climatic niche differentiation between *Fragaria* diploids and tetraploids has been explored and confirmed [30, 31], but insight into how tetraploid genomes are differentiated across these environments has not previously been explored. By comparing the functions of genes with expansion or contraction and determining the differentially expressed genes between the tetraploids and their closest diploid relative, we found that both *F. corymbosa* and *F. moupinensis* showed less expression of defense responses towards biotic stresses (such as defense response to fungus and bacterium) but in contrast has stronger expression in UV-B response, DNA repairing, and the processes of cell division (Figs 4 and 5). These trade-offs can be explained by the habitat preference of these two tetraploids [31]. The environments in high-altitude floras near QTP are characterized by freezing temperatures, high UV radiation, and hypoxia, thus plants have to evolve suites of morphologic and genetic adaptations to ensure their survival and reproduction (e.g. [37, 38]). Despite hostile environments, the biotic stresses such as herbivory or pathogen infections are much reduced in high altitudes compared to that in low-altitude habitats [39–42]. A study focusing on high-altitude adaptations showed disease-resistance genes were contracted and UV radiation-related genes were expanded in alpine plants than their low-altitude relatives, which is similar to what is found in the present study [43]. In the future, we recommend deeper research harnessing the power of genome-wide association studies (GWAS) or the determination of quantitative trait loci (QTL) to better link specific adaptation-related traits to their genetic basis.

To sum up, though introgressions may occur between diploid relatives, our results firmly support the ancestor–descendant relationship between *F. chinensis* and *F. corymbosa*, and the close evolutionary affinity between *F. chinensis* and *F. moupinensis*. The two tetraploids show similar genetic responses in high-altitude environments compared to their diploid ancestor, providing evidence of niche differentiation between polyploids and their diploid relatives in the genetic aspect.

## Materials and methods

### Plant material, genome sequencing, assembly, and annotation

To gain insights into the evolution of *F. corymbosa* and *F. moupinensis*, we sequenced and *de novo* assembled the genomes of the two tetraploids along with three *Fragaria* diploids (*F. chinensis*, *F. pentaphylla*, and *F. daltoniana*) (Table 1 and Fig. 1a). The seeds of these plants were collected from the wild (Table 1) and then germinated in a walk-in growth chamber with conditions of 22°C during 16 h of daytime and 15°C during 8 h of nighttime, with a relative humidity of 90%. After the seed germination, the seedlings were grown in a greenhouse with a temperature of 20–25°C at Taizhou University (Zhejiang, China). For the two tetraploids, we counted the chromosome number following Nathewet *et al.* [44] to confirm the ploidy of plant materials prior to genome sequencing. About 2 cm root tips were harvested around 10 a.m. and pretreated with 2 mM 8-hydroxyquinoline at 22°C for 1 h and then kept at 4°C for over 15 h. After being cleaned with distilled water, the root tips were fixed in the Farmer’s solution (absolute ethanol:glacial acetic acid = 3:1) for 2 h. Then, the root tips were soaked in 1 N HCl at 22°C for 1 h, and macerated in 1 N HCl at 60°C for 11 min. Later, the root tip was moved onto a glass slide with one drop of carbol-fuchsin staining solution and stood for 15 min. After the root tips were broken into invisible particles, the chromosomes were observed using a Motic BA310 light microscope at 100× magnification.

We assembled the genomes using PacBio SMRT long reads (20-kb fragment size) (>150×; with an average of 189×), 150-bp paired-end Illumina short reads (>100×; with an average of 244×), and Hi-C library data (>100×; with an average of 144×) (Table S6, see online supplementary material). Detailed information on DNA isolation, RNA extraction, and sequencing procedure is available in the supplementary material. We conducted a genome survey by analysing the *k*-mer frequency [45]. High-quality pair-end Illumina reads were used to generate 17-mer frequency information. The genomes were initially *de novo* assembled using the PacBio SMRT long reads with FALCON (for diploids) and FALCON-Unzip (for tetraploids) [46]. Then, the primary contigs were self-corrected with the long reads using Quiver (a toolkit in FALCON) and subsequently corrected with the Illumina short reads using NextPolish (v. 1.3.1) [47]. The Hi-C sequencing data were aligned to the draft genome using Bowtie 2 (v. 2.4.5) [48]. Then, the chromosome scaffolding was conducted with the ALLHiC (v. 0.9.8) pipeline (pruning, partitioning, rescuing, optimization, and building; —minREs 50 —maxlinkdensity 3 —NonInformativeRabio 2) [49]. For the tetraploids, four sets of pseudo-chromosomes were anchored to explore the genomic features, and one set of pseudo-chromosomes was anchored for the three diploids following the literature [26, 28, 32, 35]. Later, the chromosome-level assembly was manually corrected using Juicebox (v. 1.5) [50]. The completeness of the assembled genome was evaluated using the Benchmarking Universal Single-Copy Orthologs (BUSCO; v. 4.1.2) [51]. Finally, we annotated these genomes (including repeats, genes, and non-coding RNA genes) via a combination of homology-based inference, *de novo* prediction, and transcripts from the RNA sequencing of different tissues (leaf, root, stolon, and stem; Table S6, see online supplementary material). The detailed procedures of RNA sequencing and genome annotation are reported in the supplementary material.

### Testing the presence of subgenomes

Given that we lacked clear knowledge on whether sequenced accessions of *F. corymbosa* and *F. moupinensis* were autotetraploids or not, we tested the presence of subgenomes by detecting gene synteny, analysing *k*-mer spectrum, and analysing TE profiles. Gene synteny within the genomes of the two tetraploids was analysed using blastp (v. 2.13.0+; −evalue 1e^−5^ −num_alignments 20) and MCScanX [52] with default settings. Using GenomeScope (v. 2.0) [53], we explored how *k*-mer frequencies were distributed in the two tetraploid genomes with genome-survey data (Table S6, see online supplementary material). For autotetraploid, we would expect a high proportion of *aaab* and a low proportion of *aabb* [53]. Also, it is well-accepted that TE profiles can vary between homoeologous chromosomes when subgenomes are present (e.g. [54]). Hence, we built a summary matrix of the copy number of each TE family among pseudo-chromosomes and then performed a PCA using R package stats (v. 4.1.2) [55]. Furthermore, the results of the phylogenetic analysis could provide additional evidence for the presence or absence of subgenomes (see below).

### Identifying the diploid ancestors

We inferred the potential diploid ancestors of the two *Fragaria* tetraploids using two complementary methods: the ‘tree-based’ method and the ‘sequence-based’ method [21].

### The ‘tree-based’ method

To explore the affinity between the two tetraploids and their relatives, we inferred the phylogenetic tree based on the protein sequence data of 19 species (including 11 *Fragaria* spp.; 25 haplotypes in total) whose whole-genome information is available. Besides the five sequenced species in the present study, we included six *Fragaria* species, i.e., *F. iinumae* [20], *Fragaria mandshurica* [32], *Fragaria nilgerrensis* [32], *F. nubicola* [21], *F. vesca* [26], and *Fragaria viridis* [32]*,* seven Rosaceae species, i.e., *R. chinensis* [56], *R. occidentalis* [57], *Gillenia trifoliata* [58], *Malus domestica* [59], *Prunus mume* [60], *Prunus persica* [61], *Pyrus communis* [62], and *Arabidopsis thaliana* (v. Araport11, as the outgroup [63]) in phylogenetic analysis. We searched OGs among these taxa using OrthoFinder (v. 2.5.4) [64] with the DIAMOND search program [65]. Additionally, we searched OGs among *Fragaria* species and the two closest Rosaceae species (*R. chinensis* and *R. occidentalis*; see Results) to include more OGs for phylogenetic inferences.

To compare the robustness of reconstructed phylogeny, we generated six single-copy OG datasets with the inclusion of different species: (i) 19 species (25 haplotypes) and (ii) 13 species (19 haplotypes), and different haplotypes coverages: (i) present at no less than four haplotypes, maximizing phylogenetic information; (ii) present at more than half of the haplotypes; and (iii) present at all haplotypes. For each OG dataset, we inferred the phylogenetic tree using both the concatenation method and the coalescent-based method. The protein sequences of each OG were aligned using MAFFT (v. 7.508; —auto) [66] and then being trimmed using trimAl (v. 1.4; −automated1) [67]. For the concatenation method, the consensus maximum likelihood (ML) phylogenetic tree was inferred using IQ-TREE (v. 2.2.0.3) with 1000 ultra-fast bootstrap replicates [68, 69]. The concatenated sequences were partitioned according to OG positions. The best-fit protein substitution model was determined automatically with ModelFinder as supported by IQ-TREE (−m MFP) [70]. For the coalescent-based method, the consensus ML tree of each OG was inferred with the identical approach as described above. Then, we summarized the species tree based on gene ML trees and calculated the LPP of each node using ASTRAL (v. 5.7.8) with default settings [71].

### The ‘sequence-based’ method

As a complementary method to identify the ancestors of the two tetraploids, we applied the sppIDer approach which maps short-read sequencing data to a mixed reference genome constructed from potential ancestors to determine their contributions to hybrid genomes [72]. Here, we mapped whole-genome resequencing data of 20 tetraploid plant individuals (ten of *F. corymbosa* and *F. moupinensis* each; Table S7, see online supplementary material) to a composite genome constituted by nine *Fragaria* diploids, i.e., *F. chinensis*, *F. pentaphylla*, *F. daltoniana*, *F. iinumae*, *F. mandshurica*, *F. nilgerrensis*, *F. nubicola*, *F. vesca*, and *F. viridis*. The genome resequencing data were compiled from both the newly sequenced accessions (the same sequencing methods of the genome survey described above) and the NCBI SRA database. The raw reads downloaded from the SRA database were filtered into clean data using fastp (v. 0.23.4) with default settings [73]. The chromosome-level genome sequences of these diploids were obtained from the same sources of the protein sequences as described in the ‘tree-based’ method part. Then, we applied one-way ANOVA and post-hoc analysis (Tukey’s HSD) using the R package stats to test the significance of the difference in genome contribution among species. Further, we tested the contribution of hybrid genomes to the genome of *F. chinensis* with a similar method (see Results; Table S7, see online supplementary material).

### Genetic admixtures

To further explore introgressions among the two tetraploids and their close relatives (six species, 49 individuals; Table S7, see online supplementary material), we adopted the *D*-statistics (also known as ABBA-BABA tests) as implemented in AdmixTools (v. 7.0.2) [74]. Assuming W, X, Y, and Z are four species with a certain phylogeny (((W,X),Y),Z), W and X being the focal species, Y being the tested species potentially introgressing with W or X, and Z being the outgroup used to polarize variants (here, *F. nilgerrensis*). If W and X share equal amounts of alleles with Y, the *D*-statistic will be zero, or at least the absolute *Z*-score will be no greater than three. If the *D*-statistic is positive and *Z*-score >3, W and Y would show excess allele sharing than expected, thus implying the presence of introgression [74]. We generated SNP data as the input of AdmixTools analysis with the following steps. First, we mapped and aligned clean reads to the reference genome using BWA (v. 0.7.17-r1188) [75] and samtools (v. 1.9) [76]. Then, we performed variant calling with bcftools (v. 1.16) [76, 77]. The variants were first hard-filtered with advised parameters to remove low-quality loci and to reduce calling bias (−e 'QUAL < 20 || DP > 250 || MQBZ < −3 || RPBZ < −3 || RPBZ >3 || SCBZ >6′; https://www.htslib.org/workflow/filter.html). Then, soft-filtering was performed using vcftools (v. 0.1.16) [78]. Finally, only bi-allelic SNP loci that are present in more than 60% of individuals were retained (—max-missing 0.6 —max-alleles 2 —min-alleles 2).

### Gene family expansion and contraction

To explore genes that responded strongly during polyploidization, we inferred the changes in the gene family size of OGs (identified from the OrthoFinder analysis as described above) along the evolutionary time using CAFE (v. 5.0 [79]). The ultrametric tree was inferred based on the consensus ML tree using r8s (v. 1.81; smoothing = 0.01, divtime method = PL, algorithm = TN) [80]. The crown age of the genus *Fragaria* was set to 6.37 million years ago (mya) following Feng *et al.* [21, 28]. To account for the rate variation among OG families, we compared the likelihood through a discrete gamma distribution (*K* = 2 to *K* = 6) and then chose the best model. Later, we extracted protein sequences of OGs that exclusively expanded or contracted observed in the two tetraploids rather than in their closest diploid relatives using SeqKit (v. 2.4.0) [81]. Subsequently, we annotated these genes by querying against *A. thaliana* and then enriched them to test whether these genes were statistically strongly associated with the GO terms. The annotation and enrichment analysis were conducted using KOBAS-i [82]. The GO terms under the ‘biological process’ category were focused and only those with Fisher’s exact test (with Benjamini and Hochberg FDR correction) *p*-value <0.05 were kept (hereafter).

### Differentially expressed genes between tetraploids and their relatives

To explore whether the pattern of DEGs was partly correlated with gene family expansions or contractions between the tetraploids and their relatives, we used RNA sequencing data generated in aiding genome annotation (four tissues; Table S6, see online supplementary material) to investigate DEGs between *F. corymbosa* and *F. chinensis*, and between *F. moupinensis* and *F. chinensis*. Clean reads were mapped to the primary haplotype of each tetraploid respectively using HISAT2 (v. 2.2.1) [83]. Then, we analysed the expression levels of genes using StringTie (v. 2.2.1) [84]. The read count information was extracted using the prepDE.py script available in StringTie. Next, the DEGs were identified using the R package DESeq2 (v. 1.34.0) [85] based on the criteria of |log_2_FC| > 1 and *p*_adj_ < 0.05. Subsequently, these putative DEGs were annotated and enriched against *A. thaliana* using KOBAS-i. The GO terms under the ‘biological process’ category were focused.

## Supplementary Material

Web_Material_uhae194

## Data Availability

The assembled genome sequences and annotation files are available from the figshare repository (https://doi.org/10.6084/m9.figshare.25999996).
