# Supplementary material for: Genomic data provides insights into the evolutionary history and adaptive differentiation of two tetraploid strawberries
Source: Hortic Res. 2024 Jul 11;11(9):uhae194. doi: 10.1093/hr/uhae194 (PMC11384118; doi:10.1093/hr/uhae194)
Supplement: Web_Material_uhae194 [file web_material_uhae194.zip › Supplementary Materials_R2.pdf]

# Supplementary Methods

## DNA extraction, RNA extraction, and sequencing

Genomic DNA was isolated from mature leaves using the DNAsecure Plant Kit (TIANGEN Biotech, Beijing, China). To conduct the genome survey and the subsequent error correction, 150-bp paired-end reads of insert size 350 bp were generated using the Illumina HiSeq X Ten platform (Illumina, San Diego, CA, USA). PacBio SMRT libraries of 20-kb fragment size were constructed using the high molecular weight DNA, based on the protocol of SMRTbell library construction (https://www.pacb.com/support/documentation/), and then sequenced on the PacBio Sequel platform (PacBio, San Diego, CA, USA). Meanwhile, Hi-C libraries were prepared and sequenced using the Illumina HiSeq X Ten platform for the assembly of chromosome-level genomes. The data size of PacBio SMRT long reads (> 150×; with an average of 189×), Illumina short reads (> 100×; with an average of 244×), and Hi-C libraries (> 100×; with an average of 144×) generated for the genome survey, assembly, error correction, and Hi-C scaffolding of each accession are available in Table S1. To aid genome annotation, we performed RNA sequencing (RNA-seq). Total RNA was extracted from the leaf, root, stolon, and stem using the RNAprep Pure Plant Kit (TIANGEN Biotech), and then the genomic DNA contamination was removed using RNase-Free DNase I (TIANGEN Biotech). The integrity of RNA was evaluated on a 1.0% agarose gel stained with ethidium bromide, and its quality and quantity were assessed using the Agilent 2100 Bioanalyzer (Agilent Technologies, Santa Clara, CA, USA). The cDNA libraries were constructed using the NEBNext Ultra RNA Library Prep Kit for Illumina (New England Biolabs, Ipswich, MA, USA), following the manufacturer's protocol, and then RNA-seq was performed on the Illumina HiSeq X Ten platform. Raw paired-end reads of Illumina sequencing were processed to remove adapters and low-quality sequences (the unknown base ratio was higher than 10% and the low-quality base ratio [Q < = 5] was more than 20%) at the commercial sequencing institution (Novogene, Beijing, China).

## Genome annotation

Whole genome repeats were identified using *de novo* search and homology alignment. We extracted tandem repeats using TRF (v. 4.09) (Benson, 1999) with *ab initio* prediction. We used the homolog prediction in the Repbase database (Jurka, 2000) by employing RepeatMasker (v. 4.1.0) (Tarailo-Graovac, & Chen, 2009) and its in-house scripts (RepeatProteinMask) with default parameters to extract repeat regions. We used *ab initio* predictions with default parameters in LTR_FINDER (v. 1.0.6) (Xu & Wang, 2007), RepeatScout (v. 1.0.5) (Prize et al., 2005), and RepeatModeler (v. 2.0.1) (Flynn et al., 2020) to build a *de novo* database of repetitive elements. All repeat sequences with lengths >100 bp and gap 'N' less than 5% constituted the library of raw transposable elements (TEs). A combination of repeat regions from Repbase and our *de novo* TE library was processed by UCLUST (Edgar, 2010) to yield a non-redundant library, which was then supplied to RepeatMasker to identify DNA-level repeats. To annotate the genes of the five *Fragaria* species, a combination of homology-based inference, *de novo* prediction, and transcripts from the RNA-seq was used. For homology-based prediction, protein sequences of five Rosaceae species: *Fragaria vesca* (Shulaev et al., 2011), *Malus domestica* (Daccord et al., 2017), *Prunus persica* (Verde et al., 2013), *Prunus mume* (Zhang et al., 2012), and *Pyrus bretschneideri* (Wu et al., 2013) were mapped to the repeat-masked genome with tBLASTn (v. 2.2.26; E-value $\leq 10^{-5}$) (Altschul et al., 1990). The matching proteins were then aligned with homologous genome sequences with GeneWise (v. 2.4.1) (Birney et al., 2004). For gene prediction based on *ab initio* methods, we used AUGUSTUS (v. 3.2.3) (Stanke et al., 2008), geneid (v. 1.4) (Blanco et al., 2007), GENSCAN (v. 1.0) (Burge & Karlin, 1997), GlimmerHMM (v.3 .0.4) (Majoros et al., 2004), and SNAP (v. 2013-11-29) (Korf, 2004) in the automated gene prediction pipeline. For RNA-seq data, reads were initially assembled with Trinity (v. 2.1.1) (Grabherr et al., 2011). To optimize the annotation, assembled reads from different tissues were aligned with the genome by HISAT (v. 2.1.0) (Kim et al., 2019). The alignments were then used as inputs for StringTie (v. 1.3.3) (Pertea et al., 2015) to generate genome-based transcript assemblies. Finally, we generated a non-redundant reference gene set by merging genes predicted by the three methods with EVidenceModeler (v. 1.1.1) and PASA (Haas et al., 2008).

## References


Altschul, S. F., Gish, W., Miller, W., Myers, E. W., & Lipman, D. J. (1990). Basic local alignment search tool. *Journal of Molecular Biology*, *215*(3), 403–410. https://doi.org/10.1016/S0022-2836(05)80360-2

Benson, G. (1999). Tandem repeats finder: a program to analyze DNA sequences. *Nucleic Acids Research*, *27*(2), 573–580. https://doi.org/10.1093/nar/27.2.573

Birney, E., Clamp, M., & Durbin, R. (2004). GeneWise and Genomewise. *Genome Research*, *14*(5), 988–995. https://doi.org/10.1101/gr.1865504

Blanco, E., Parra, G., & Guigó, R. (2007). Using geneid to Identify Genes. *Current Protocols in Bioinformatics*, *18*(1), 4.3. https://doi.org/10.1002/0471250953.bi0403s18

Burge, C., & Karlin, S. (1997). Prediction of complete gene structures in human genomic DNA. *Journal of Molecular Biology*, *268*(1), 78–94. https://doi.org/10.1006/jmbi.1997.0951

Daccord, N., Celton, J.-M., Linsmith, G., Becker, C., Choisne, N., Schijlen, E., van de Geest, H., Bianco, L., Micheletti, D., Velasco, R., Di Pierro, E. A., Gouzy, J., Rees, D. J. G., Guérif, P., Muranty, H., Durel, C.-E., Laurens, F., Lespinasse, Y., Gaillard, S., … Bucher, E. (2017). High-quality de novo assembly of the apple genome and methylome dynamics of early fruit development. *Nature Genetics*, *49*(7), 1099–1106. https://doi.org/10.1038/ng.3886

Edgar, R. C. (2010). Search and clustering orders of magnitude faster than BLAST. *Bioinformatics*, *26*(19), 2460–2461. https://doi.org/10.1093/bioinformatics/btq461

Flynn, J. M., Hubley, R., Goubert, C., Rosen, J., Clark, A. G., Feschotte, C., & Smit, A. F. (2020). RepeatModeler2 for automated genomic discovery of transposable element families. *Proceedings of the National Academy of Sciences*, *117*(17), 9451–9457. https://doi.org/10.1073/pnas.1921046117

Grabherr, M. G., Haas, B. J., Yassour, M., Levin, J. Z., Thompson, D. A., Amit, I., Adiconis, X., Fan, L., Raychowdhury, R., Zeng, Q., Chen, Z., Mauceli, E., Hacohen, N., Gnirke, A., Rhind, N., di Palma, F., Birren, B. W., Nusbaum, C., Lindblad-Toh, K., … Regev, A. (2011). Full-length transcriptome assembly from RNA-Seq data without a reference genome. *Nature Biotechnology*, *29*(7), 644–652. https://doi.org/10.1038/nbt.1883

Haas, B. J., Salzberg, S. L., Zhu, W., Pertea, M., Allen, J. E., Orvis, J., White, O., Buell, C. R., & Wortman, J. R. (2008). Automated eukaryotic gene structure annotation using EVidenceModeler and the Program to Assemble Spliced Alignments. *Genome Biology*, *9*(1), R7. https://doi.org/10.1186/gb-2008-9-1-r7

Jurka, J. (2000). Repbase Update: a database and an electronic journal of repetitive elements. *Trends in Genetics*, *16*(9), 418–420. https://doi.org/10.1016/S0168-9525(00)02093-X

Kim, D., Paggi, J. M., Park, C., Bennett, C., & Salzberg, S. L. (2019). Graph-based genome alignment and genotyping with HISAT2 and HISAT-genotype. *Nature Biotechnology*, *37*(8), 907–915. https://doi.org/10.1038/s41587-019-0201-4

Korf, I. (2004). Gene finding in novel genomes. *BMC Bioinformatics*, *5*, 59. https://doi.org/10.1186/1471-2105-5-59

Majoros, W. H., Pertea, M., & Salzberg, S. L. (2004). TigrScan and GlimmerHMM: two open source ab initio eukaryotic gene-finders. *Bioinformatics*, *20*(16), 2878–2879. https://doi.org/10.1093/bioinformatics/bth315

Pertea, M., Pertea, G. M., Antonescu, C. M., Chang, T.-C., Mendell, J. T., & Salzberg, S. L. (2015). StringTie enables improved reconstruction of a transcriptome from RNA-seq reads. *Nature Biotechnology*, *33*(3), 290–295. https://doi.org/10.1038/nbt.3122

Price, A. L., Jones, N. C., & Pevzner, P. A. (2005). De novo identification of repeat families in large genomes. *Bioinformatics*, *21*, i351–i358. https://doi.org/10.1093/bioinformatics/bti1018

Shulaev, V., Sargent, D. J., Crowhurst, R. N., Mockler, T. C., Folkerts, O., Delcher, A. L., Jaiswal, P., Mockaitis, K., Liston, A., Mane, S. P., Burns, P., Davis, T. M., Slovin, J. P., Bassil, N., Hellens, R. P., Evans, C., Harkins, T., Kodira, C., Desany, B., … Folta, K. M. (2011). The genome of woodland strawberry (Fragaria vesca). *Nature Genetics*, *43*(2), 109–116. https://doi.org/10.1038/ng.740

Stanke, M., Diekhans, M., Baertsch, R., & Haussler, D. (2008). Using native and syntenically mapped cDNA alignments to improve de novo gene finding. *Bioinformatics*, *24*(5), 637–644. https://doi.org/10.1093/bioinformatics/btn013

Tarailo-Graovac, M., & Chen, N. (2009). Using RepeatMasker to Identify Repetitive Elements in Genomic Sequences. *Current Protocols in Bioinformatics*, *25*(1), 4.10.1-4.10.14. https://doi.org/10.1002/0471250953.bi0410s25

Verde, I., Abbott, A. G., Scalabrin, S., Jung, S., Shu, S., Marroni, F., Zhebentyayeva, T., Dettori, M. T., Grimwood, J., Cattonaro, F., Zuccolo, A., Rossini, L., Jenkins, J., Vendramin, E., Meisel, L. A., Decroocq, V., Sosinski, B., Prochnik, S., Mitros, T., … Rokhsar, D. S. (2013). The high-quality draft genome of peach (Prunus persica) identifies unique patterns of genetic diversity, domestication and genome evolution. *Nature Genetics*, *45*(5), 487–494. https://doi.org/10.1038/ng.2586

Wu, J., Wang, Z., Shi, Z., Zhang, S., Ming, R., Zhu, S., Khan, M. A., Tao, S., Korban, S. S., Wang, H., Chen, N. J., Nishio, T., Xu, X., Cong, L., Qi, K., Huang, X., Wang, Y., Zhao, X., Wu, J., … Zhang, S. (2013). The genome of the pear ( Pyrus bretschneideri Rehd.). *Genome Research*, *23*(2), 396–408. https://doi.org/10.1101/gr.144311.112

Xu, Z., & Wang, H. (2007). LTR_FINDER: an efficient tool for the prediction of full-length LTR retrotransposons. *Nucleic Acids Research*, *35*, W265–W268. https://doi.org/10.1093/nar/gkm286

Zhang, Q., Chen, W., Sun, L., Zhao, F., Huang, B., Yang, W., Tao, Y., Wang, J., Yuan, Z., Fan, G., Xing, Z., Han, C., Pan, H., Zhong, X., Shi, W., Liang, X., Du, D., Sun, F., Xu, Z., … Wang, J. (2012). The genome of Prunus mume. *Nature Communications*, *3*(1), 1318. https://doi.org/10.1038/ncomms2290

# Supplementary Tables

Table S1 Mapping rate of individuals to composite genomes in the sppIDer analysis

| *Fragaria* species | Accession ID | Ancestor | Reads count | Mapping percentage |
|---|---|---|---|---|
| *F. corymbosa* | FDES192246202 | Fch | 35197293 | 48.42% |
| *F. corymbosa* | FDES192246202 | Fnu | 13831598 | 16.68% |
| *F. corymbosa* | FDES192246202 | Fpe | 19441914 | 23.97% |
| *F. corymbosa* | FDES192246202 | Fda | 3566239 | 3.71% |
| *F. corymbosa* | FDES192246202 | Fii | 1222727 | 1.27% |
| *F. corymbosa* | FDES192246202 | Fma | 1495309 | 1.01% |
| *F. corymbosa* | FDES192246202 | Fni | 2446187 | 2.67% |
| *F. corymbosa* | FDES192246202 | Fvi | 1579300 | 1.57% |
| *F. corymbosa* | FDES192246202 | Fve | 1188921 | 0.71% |
| *F. corymbosa* | FDES192246204 | Fch | 26609195 | 41.38% |
| *F. corymbosa* | FDES192246204 | Fnu | 13301061 | 18.67% |
| *F. corymbosa* | FDES192246204 | Fpe | 17125067 | 24.29% |
| *F. corymbosa* | FDES192246204 | Fda | 3797876 | 4.65% |
| *F. corymbosa* | FDES192246204 | Fii | 1388476 | 1.71% |
| *F. corymbosa* | FDES192246204 | Fma | 2527173 | 2.26% |
| *F. corymbosa* | FDES192246204 | Fni | 2541954 | 3.21% |
| *F. corymbosa* | FDES192246204 | Fvi | 1868574 | 2.36% |
| *F. corymbosa* | FDES192246204 | Fve | 1851823 | 1.47% |
| *F. corymbosa* | SCH362D | Fch | 9950006 | 32.37% |
| *F. corymbosa* | SCH362D | Fnu | 6445287 | 20.56% |
| *F. corymbosa* | SCH362D | Fpe | 10081519 | 33.96% |
| *F. corymbosa* | SCH362D | Fda | 1804935 | 4.72% |
| *F. corymbosa* | SCH362D | Fii | 643892 | 1.68% |
| *F. corymbosa* | SCH362D | Fma | 663246 | 1.10% |

| | | | | |
|---|---|---|---|---|
| *F. corymbosa* | SCH362D | Fni | 1124510 | 3.15% |
| *F. corymbosa* | SCH362D | Fvi | 727544 | 1.75% |
| *F. corymbosa* | SCH362D | Fve | 462390 | 0.70% |
| *F. corymbosa* | SRR5275236 | Fch | 628431 | 48.62% |
| *F. corymbosa* | SRR5275236 | Fnu | 346493 | 15.94% |
| *F. corymbosa* | SRR5275236 | Fpe | 380718 | 19.64% |
| *F. corymbosa* | SRR5275236 | Fda | 109773 | 3.81% |
| *F. corymbosa* | SRR5275236 | Fii | 74181 | 4.25% |
| *F. corymbosa* | SRR5275236 | Fma | 56599 | 1.32% |
| *F. corymbosa* | SRR5275236 | Fni | 80996 | 3.10% |
| *F. corymbosa* | SRR5275236 | Fvi | 56634 | 2.40% |
| *F. corymbosa* | SRR5275236 | Fve | 47052 | 0.92% |
| *F. corymbosa* | SRR11788127 | Fch | 15152947 | 51.28% |
| *F. corymbosa* | SRR11788127 | Fnu | 6530202 | 15.02% |
| *F. corymbosa* | SRR11788127 | Fpe | 8257167 | 20.36% |
| *F. corymbosa* | SRR11788127 | Fda | 2260368 | 4.38% |
| *F. corymbosa* | SRR11788127 | Fii | 922467 | 1.71% |
| *F. corymbosa* | SRR11788127 | Fma | 1075245 | 1.08% |
| *F. corymbosa* | SRR11788127 | Fni | 1684239 | 3.32% |
| *F. corymbosa* | SRR11788127 | Fvi | 1071934 | 2.05% |
| *F. corymbosa* | SRR11788127 | Fve | 823497 | 0.80% |
| *F. corymbosa* | SRR13775102 | Fch | 84881602 | 49.33% |
| *F. corymbosa* | SRR13775102 | Fnu | 31610742 | 15.51% |
| *F. corymbosa* | SRR13775102 | Fpe | 41074521 | 20.40% |
| *F. corymbosa* | SRR13775102 | Fda | 11809551 | 5.01% |
| *F. corymbosa* | SRR13775102 | Fii | 4705971 | 1.95% |
| *F. corymbosa* | SRR13775102 | Fma | 4920856 | 1.35% |
| *F. corymbosa* | SRR13775102 | Fni | 6999728 | 3.16% |
| *F. corymbosa* | SRR13775102 | Fvi | 5090247 | 2.34% |

| | | | | |
|---|---|---|---|---|
| *F. corymbosa* | SRR13775102 | Fve | 3427050 | 0.95% |
| *F. corymbosa* | SRR22800220 | Fch | 81169443 | 51.52% |
| *F. corymbosa* | SRR22800220 | Fnu | 28829044 | 15.26% |
| *F. corymbosa* | SRR22800220 | Fpe | 40884756 | 22.44% |
| *F. corymbosa* | SRR22800220 | Fda | 7787408 | 3.61% |
| *F. corymbosa* | SRR22800220 | Fii | 2702980 | 1.25% |
| *F. corymbosa* | SRR22800220 | Fma | 3372584 | 1.01% |
| *F. corymbosa* | SRR22800220 | Fni | 5363999 | 2.65% |
| *F. corymbosa* | SRR22800220 | Fvi | 3310780 | 1.53% |
| *F. corymbosa* | SRR22800220 | Fve | 2622124 | 0.72% |
| *F. corymbosa* | SRR22800221 | Fch | 84429945 | 51.26% |
| *F. corymbosa* | SRR22800221 | Fnu | 30414449 | 15.42% |
| *F. corymbosa* | SRR22800221 | Fpe | 42382134 | 22.05% |
| *F. corymbosa* | SRR22800221 | Fda | 8517039 | 3.73% |
| *F. corymbosa* | SRR22800221 | Fii | 3108981 | 1.39% |
| *F. corymbosa* | SRR22800221 | Fma | 3621954 | 1.02% |
| *F. corymbosa* | SRR22800221 | Fni | 5796548 | 2.71% |
| *F. corymbosa* | SRR22800221 | Fvi | 3893373 | 1.69% |
| *F. corymbosa* | SRR22800221 | Fve | 2761170 | 0.74% |
| *F. corymbosa* | SRR22800223 | Fch | 92395909 | 51.84% |
| *F. corymbosa* | SRR22800223 | Fnu | 32119515 | 15.25% |
| *F. corymbosa* | SRR22800223 | Fpe | 45270191 | 22.35% |
| *F. corymbosa* | SRR22800223 | Fda | 8383250 | 3.48% |
| *F. corymbosa* | SRR22800223 | Fii | 2938156 | 1.24% |
| *F. corymbosa* | SRR22800223 | Fma | 3730580 | 1.01% |
| *F. corymbosa* | SRR22800223 | Fni | 5864265 | 2.59% |
| *F. corymbosa* | SRR22800223 | Fvi | 3544778 | 1.50% |
| *F. corymbosa* | SRR22800223 | Fve | 3015149 | 0.73% |
| *F. corymbosa* | SRR22800224 | Fch | 88575103 | 52.01% |

| | | | | |
|---|---|---|---|---|
| *F. corymbosa* | SRR22800224 | Fnu | 31378044 | 15.23% |
| *F. corymbosa* | SRR22800224 | Fpe | 44815867 | 22.53% |
| *F. corymbosa* | SRR22800224 | Fda | 7807321 | 3.33% |
| *F. corymbosa* | SRR22800224 | Fii | 2772337 | 1.22% |
| *F. corymbosa* | SRR22800224 | Fma | 3516277 | 0.95% |
| *F. corymbosa* | SRR22800224 | Fni | 5584206 | 2.51% |
| *F. corymbosa* | SRR22800224 | Fvi | 3291360 | 1.48% |
| *F. corymbosa* | SRR22800224 | Fve | 2852612 | 0.73% |
| *F. moupinensis* | FDES192246207 | Fch | 25506418 | 34.60% |
| *F. moupinensis* | FDES192246207 | Fnu | 16985161 | 22.35% |
| *F. moupinensis* | FDES192246207 | Fpe | 22889870 | 30.87% |
| *F. moupinensis* | FDES192246207 | Fda | 3787948 | 4.13% |
| *F. moupinensis* | FDES192246207 | Fii | 1271423 | 1.41% |
| *F. moupinensis* | FDES192246207 | Fma | 1571045 | 1.11% |
| *F. moupinensis* | FDES192246207 | Fni | 2636843 | 3.02% |
| *F. moupinensis* | FDES192246207 | Fvi | 1610154 | 1.70% |
| *F. moupinensis* | FDES192246207 | Fve | 1254825 | 0.80% |
| *F. moupinensis* | GSH454B | Fch | 30105084 | 36.01% |
| *F. moupinensis* | GSH454B | Fnu | 23394902 | 28.25% |
| *F. moupinensis* | GSH454B | Fpe | 20446633 | 22.71% |
| *F. moupinensis* | GSH454B | Fda | 4954406 | 4.69% |
| *F. moupinensis* | GSH454B | Fii | 1680897 | 1.60% |
| *F. moupinensis* | GSH454B | Fma | 1853723 | 1.12% |
| *F. moupinensis* | GSH454B | Fni | 3054966 | 3.02% |
| *F. moupinensis* | GSH454B | Fvi | 1888426 | 1.78% |
| *F. moupinensis* | GSH454B | Fve | 1407448 | 0.82% |
| *F. moupinensis* | GSH494B | Fch | 38964886 | 49.70% |
| *F. moupinensis* | GSH494B | Fnu | 14309618 | 15.51% |
| *F. moupinensis* | GSH494B | Fpe | 19339474 | 21.19% |

| | | | | |
|---|---|---|---|---|
| *F. moupinensis* | GSH494B | Fda | 5234761 | 4.88% |
| *F. moupinensis* | GSH494B | Fii | 2028170 | 1.89% |
| *F. moupinensis* | GSH494B | Fma | 1954169 | 1.16% |
| *F. moupinensis* | GSH494B | Fni | 2986800 | 2.88% |
| *F. moupinensis* | GSH494B | Fvi | 2206315 | 1.98% |
| *F. moupinensis* | GSH494B | Fve | 1361467 | 0.81% |
| *F. moupinensis* | QHH454A | Fch | 37048114 | 46.63% |
| *F. moupinensis* | QHH454A | Fnu | 18839955 | 22.29% |
| *F. moupinensis* | QHH454A | Fpe | 18219890 | 19.99% |
| *F. moupinensis* | QHH454A | Fda | 4306744 | 4.00% |
| *F. moupinensis* | QHH454A | Fii | 1494395 | 1.38% |
| *F. moupinensis* | QHH454A | Fma | 1577497 | 0.92% |
| *F. moupinensis* | QHH454A | Fni | 2598982 | 2.53% |
| *F. moupinensis* | QHH454A | Fvi | 1813160 | 1.62% |
| *F. moupinensis* | QHH454A | Fve | 1173658 | 0.65% |
| *F. moupinensis* | SCH454B | Fch | 34538024 | 36.88% |
| *F. moupinensis* | SCH454B | Fnu | 18169584 | 18.27% |
| *F. moupinensis* | SCH454B | Fpe | 28807898 | 30.68% |
| *F. moupinensis* | SCH454B | Fda | 6244518 | 5.21% |
| *F. moupinensis* | SCH454B | Fii | 2400721 | 1.98% |
| *F. moupinensis* | SCH454B | Fma | 2163688 | 1.10% |
| *F. moupinensis* | SCH454B | Fni | 3610646 | 3.15% |
| *F. moupinensis* | SCH454B | Fvi | 2542598 | 1.96% |
| *F. moupinensis* | SCH454B | Fve | 1516646 | 0.77% |
| *F. moupinensis* | SCH574D | Fch | 27255127 | 35.28% |
| *F. moupinensis* | SCH574D | Fnu | 17478865 | 21.77% |
| *F. moupinensis* | SCH574D | Fpe | 22954174 | 29.30% |
| *F. moupinensis* | SCH574D | Fda | 4651912 | 4.82% |
| *F. moupinensis* | SCH574D | Fii | 1658158 | 1.71% |

| | | | | |
|---|---|---|---|---|
| *F. moupinensis* | SCH574D | Fma | 1783787 | 1.16% |
| *F. moupinensis* | SCH574D | Fni | 2984187 | 3.24% |
| *F. moupinensis* | SCH574D | Fvi | 1815424 | 1.86% |
| *F. moupinensis* | SCH574D | Fve | 1338543 | 0.85% |
| *F. moupinensis* | YNH634D | Fch | 27040931 | 32.22% |
| *F. moupinensis* | YNH634D | Fnu | 21446146 | 26.22% |
| *F. moupinensis* | YNH634D | Fpe | 23689377 | 28.47% |
| *F. moupinensis* | YNH634D | Fda | 4628880 | 4.51% |
| *F. moupinensis* | YNH634D | Fii | 1565799 | 1.56% |
| *F. moupinensis* | YNH634D | Fma | 1822350 | 1.13% |
| *F. moupinensis* | YNH634D | Fni | 3157075 | 3.29% |
| *F. moupinensis* | YNH634D | Fvi | 1850379 | 1.77% |
| *F. moupinensis* | YNH634D | Fve | 1400722 | 0.83% |
| *F. moupinensis* | YNH644C | Fch | 30132116 | 32.42% |
| *F. moupinensis* | YNH644C | Fnu | 23675598 | 26.00% |
| *F. moupinensis* | YNH644C | Fpe | 26078609 | 27.95% |
| *F. moupinensis* | YNH644C | Fda | 5436489 | 4.78% |
| *F. moupinensis* | YNH644C | Fii | 1841576 | 1.65% |
| *F. moupinensis* | YNH644C | Fma | 2084495 | 1.20% |
| *F. moupinensis* | YNH644C | Fni | 3584103 | 3.34% |
| *F. moupinensis* | YNH644C | Fvi | 2088060 | 1.82% |
| *F. moupinensis* | YNH644C | Fve | 1567507 | 0.84% |
| *F. moupinensis* | YNH664B | Fch | 31496456 | 32.31% |
| *F. moupinensis* | YNH664B | Fnu | 24621602 | 25.78% |
| *F. moupinensis* | YNH664B | Fpe | 26765589 | 27.32% |
| *F. moupinensis* | YNH664B | Fda | 6091422 | 5.12% |
| *F. moupinensis* | YNH664B | Fii | 2118090 | 1.81% |
| *F. moupinensis* | YNH664B | Fma | 2343013 | 1.34% |
| *F. moupinensis* | YNH664B | Fni | 3976955 | 3.54% |

| | | | | |
|---|---|---|---|---|
| *F. moupinensis* | YNH664B | Fvi | 2362712 | 1.90% |
| *F. moupinensis* | YNH664B | Fve | 1706338 | 0.89% |
| *F. moupinensis* | SCH734D | Fch | 29461661 | 44.18% |
| *F. moupinensis* | SCH734D | Fnu | 13624004 | 18.58% |
| *F. moupinensis* | SCH734D | Fpe | 18537437 | 25.84% |
| *F. moupinensis* | SCH734D | Fda | 3313619 | 3.86% |
| *F. moupinensis* | SCH734D | Fii | 1135589 | 1.35% |
| *F. moupinensis* | SCH734D | Fma | 1407458 | 1.07% |
| *F. moupinensis* | SCH734D | Fni | 2253704 | 2.73% |
| *F. moupinensis* | SCH734D | Fvi | 1385375 | 1.60% |
| *F. moupinensis* | SCH734D | Fve | 1138212 | 0.81% |
| *F. chinensis* | SAH272D | Fco | 522233 | 30.60% |
| *F. chinensis* | SAH272D | Fmo | 300384 | 17.60% |
| *F. chinensis* | SAH272D | Fnu | 231680 | 13.57% |
| *F. chinensis* | SAH272D | Fpe | 324186 | 18.99% |
| *F. chinensis* | SAH272D | Fda | 105025 | 6.15% |
| *F. chinensis* | SAH272D | Fii | 44360 | 2.60% |
| *F. chinensis* | SAH272D | Fma | 36915 | 2.16% |
| *F. chinensis* | SAH272D | Fni | 57972 | 3.40% |
| *F. chinensis* | SAH272D | Fvi | 57723 | 3.38% |
| *F. chinensis* | SAH272D | Fve | 26414 | 1.55% |
| *F. chinensis* | SAH282C | Fco | 2935527 | 30.99% |
| *F. chinensis* | SAH282C | Fmo | 1643824 | 17.36% |
| *F. chinensis* | SAH282C | Fnu | 1308610 | 13.82% |
| *F. chinensis* | SAH282C | Fpe | 1788241 | 18.88% |
| *F. chinensis* | SAH282C | Fda | 586352 | 6.19% |
| *F. chinensis* | SAH282C | Fii | 261801 | 2.76% |
| *F. chinensis* | SAH282C | Fma | 209316 | 2.21% |
| *F. chinensis* | SAH282C | Fni | 319344 | 3.37% |

| | | | | |
|---|---|---|---|---|
| *F. chinensis* | SAH282C | Fvi | 259490 | 2.74% |
| *F. chinensis* | SAH282C | Fve | 158745 | 1.68% |
| *F. chinensis* | SAH762A | Fco | 4796431 | 29.68% |
| *F. chinensis* | SAH762A | Fmo | 2720452 | 16.84% |
| *F. chinensis* | SAH762A | Fnu | 2229331 | 13.80% |
| *F. chinensis* | SAH762A | Fpe | 2926860 | 18.11% |
| *F. chinensis* | SAH762A | Fda | 1162033 | 7.19% |
| *F. chinensis* | SAH762A | Fii | 514372 | 3.18% |
| *F. chinensis* | SAH762A | Fma | 396339 | 2.45% |
| *F. chinensis* | SAH762A | Fni | 602518 | 3.73% |
| *F. chinensis* | SAH762A | Fvi | 550052 | 3.40% |
| *F. chinensis* | SAH762A | Fve | 260823 | 1.61% |
| *F. chinensis* | SAH782B | Fco | 2853013 | 31.84% |
| *F. chinensis* | SAH782B | Fmo | 1568929 | 17.51% |
| *F. chinensis* | SAH782B | Fnu | 1215886 | 13.57% |
| *F. chinensis* | SAH782B | Fpe | 1715944 | 19.15% |
| *F. chinensis* | SAH782B | Fda | 506620 | 5.65% |
| *F. chinensis* | SAH782B | Fii | 222329 | 2.48% |
| *F. chinensis* | SAH782B | Fma | 188995 | 2.11% |
| *F. chinensis* | SAH782B | Fni | 292084 | 3.26% |
| *F. chinensis* | SAH782B | Fvi | 254330 | 2.84% |
| *F. chinensis* | SAH782B | Fve | 143146 | 1.60% |
| *F. chinensis* | SRR11788122 | Fco | 5319444 | 37.64% |
| *F. chinensis* | SRR11788122 | Fmo | 2313970 | 16.37% |
| *F. chinensis* | SRR11788122 | Fnu | 1716941 | 12.15% |
| *F. chinensis* | SRR11788122 | Fpe | 2712586 | 19.19% |
| *F. chinensis* | SRR11788122 | Fda | 561163 | 3.97% |
| *F. chinensis* | SRR11788122 | Fii | 258818 | 1.83% |
| *F. chinensis* | SRR11788122 | Fma | 252016 | 1.78% |

| | | | | |
|---|---|---|---|---|
| *F. chinensis* | SRR11788122 | Fni | 380209 | 2.69% |
| *F. chinensis* | SRR11788122 | Fvi | 375698 | 2.66% |
| *F. chinensis* | SRR11788122 | Fve | 243290 | 1.72% |
| *F. chinensis* | SRR22800229 | Fco | 15735849 | 37.32% |
| *F. chinensis* | SRR22800229 | Fmo | 7102215 | 16.84% |
| *F. chinensis* | SRR22800229 | Fnu | 5274197 | 12.51% |
| *F. chinensis* | SRR22800229 | Fpe | 8691509 | 20.61% |
| *F. chinensis* | SRR22800229 | Fda | 1508648 | 3.58% |
| *F. chinensis* | SRR22800229 | Fii | 641613 | 1.52% |
| *F. chinensis* | SRR22800229 | Fma | 759629 | 1.80% |
| *F. chinensis* | SRR22800229 | Fni | 1094402 | 2.60% |
| *F. chinensis* | SRR22800229 | Fvi | 685774 | 1.63% |
| *F. chinensis* | SRR22800229 | Fve | 673575 | 1.60% |
| *F. chinensis* | SRR22800240 | Fco | 13666365 | 31.96% |
| *F. chinensis* | SRR22800240 | Fmo | 7486789 | 17.51% |
| *F. chinensis* | SRR22800240 | Fnu | 5420990 | 12.68% |
| *F. chinensis* | SRR22800240 | Fpe | 9891913 | 23.13% |
| *F. chinensis* | SRR22800240 | Fda | 1791493 | 4.19% |
| *F. chinensis* | SRR22800240 | Fii | 801105 | 1.87% |
| *F. chinensis* | SRR22800240 | Fma | 759623 | 1.78% |
| *F. chinensis* | SRR22800240 | Fni | 1193382 | 2.79% |
| *F. chinensis* | SRR22800240 | Fvi | 849302 | 1.99% |
| *F. chinensis* | SRR22800240 | Fve | 902685 | 2.11% |
| *F. chinensis* | SRR22800251 | Fco | 12502540 | 27.91% |
| *F. chinensis* | SRR22800251 | Fmo | 8459110 | 18.88% |
| *F. chinensis* | SRR22800251 | Fnu | 6399489 | 14.28% |
| *F. chinensis* | SRR22800251 | Fpe | 10765340 | 24.03% |
| *F. chinensis* | SRR22800251 | Fda | 1899134 | 4.24% |
| *F. chinensis* | SRR22800251 | Fii | 817625 | 1.83% |

| | | | | |
|---|---|---|---|---|
| *F. chinensis* | SRR22800251 | Fma | 754065 | 1.68% |
| *F. chinensis* | SRR22800251 | Fni | 1162723 | 2.60% |
| *F. chinensis* | SRR22800251 | Fvi | 822298 | 1.84% |
| *F. chinensis* | SRR22800251 | Fve | 1216371 | 2.72% |
| *F. chinensis* | SRR22800252 | Fco | 13048866 | 27.77% |
| *F. chinensis* | SRR22800252 | Fmo | 7088758 | 15.08% |
| *F. chinensis* | SRR22800252 | Fnu | 5939209 | 12.64% |
| *F. chinensis* | SRR22800252 | Fpe | 13945179 | 29.67% |
| *F. chinensis* | SRR22800252 | Fda | 2016936 | 4.29% |
| *F. chinensis* | SRR22800252 | Fii | 827527 | 1.76% |
| *F. chinensis* | SRR22800252 | Fma | 960642 | 2.04% |
| *F. chinensis* | SRR22800252 | Fni | 1444663 | 3.07% |
| *F. chinensis* | SRR22800252 | Fvi | 906276 | 1.93% |
| *F. chinensis* | SRR22800252 | Fve | 817332 | 1.74% |
| *F. chinensis* | SRR5275238 | Fco | 1671036 | 21.15% |
| *F. chinensis* | SRR5275238 | Fmo | 1378478 | 17.45% |
| *F. chinensis* | SRR5275238 | Fnu | 1232912 | 15.60% |
| *F. chinensis* | SRR5275238 | Fpe | 1489072 | 18.85% |
| *F. chinensis* | SRR5275238 | Fda | 507087 | 6.42% |
| *F. chinensis* | SRR5275238 | Fii | 306981 | 3.89% |
| *F. chinensis* | SRR5275238 | Fma | 285187 | 3.61% |
| *F. chinensis* | SRR5275238 | Fni | 387381 | 4.90% |
| *F. chinensis* | SRR5275238 | Fvi | 367972 | 4.66% |
| *F. chinensis* | SRR5275238 | Fve | 275511 | 3.49% |

Footnotes: Fch = *F. chinensis*, Fnu = *F. nubicola*, Fpe = *F. pentaphylla*, Fda = *F. daltoniana*, Fii = *F. iinumae*, Fma = *F. mandshurica*, Fni = *F. nilgerrensis*, Fvi = *F. viridis*, Fve = *F. vesca*, Fco = *F. corymbosa*, Fmo = *F. moupinensis*.

Table S2 Significant *D*-statistics among studied *Fragaria* species

| Species W | Species X | Species Y | Species Z | *D*-statistic | *Z*-score | BABA | ABBA |
|---|---|---|---|---|---|---|---|
| *F. pentaphylla* | *F. nubicola* | *F. chinensis* | *F. nilgerrensis* | 0.1889 | 24.128 | 1277 | 871 |
| *F. chinensis* | *F. nubicola* | *F. corymbosa* | *F. nilgerrensis* | 0.1734 | 16.491 | 1419 | 1000 |
| *F. corymbosa* | *F. nubicola* | *F. chinensis* | *F. nilgerrensis* | 0.1802 | 16.437 | 1419 | 986 |
| *F. moupinensis* | *F. nubicola* | *F. corymbosa* | *F. nilgerrensis* | 0.0951 | 16.29 | 1416 | 1170 |
| *F. pentaphylla* | *F. nubicola* | *F. corymbosa* | *F. nilgerrensis* | 0.1177 | 15.828 | 1307 | 1032 |
| *F. pentaphylla* | *F. moupinensis* | *F. chinensis* | *F. nilgerrensis* | 0.0936 | 15.633 | 1129 | 936 |
| *F. chinensis* | *F. nubicola* | *F. pentaphylla* | *F. nilgerrensis* | 0.1198 | 15.083 | 1277 | 1004 |
| *F. pentaphylla* | *F. nubicola* | *F. moupinensis* | *F. nilgerrensis* | 0.0672 | 13.208 | 1273 | 1113 |
| *F. corymbosa* | *F. moupinensis* | *F. chinensis* | *F. nilgerrensis* | 0.0972 | 13.025 | 1246 | 1026 |
| *F. moupinensis* | *F. nubicola* | *F. chinensis* | *F. nilgerrensis* | 0.0919 | 12.584 | 1262 | 1049 |
| *F. corymbosa* | *F. nubicola* | *F. moupinensis* | *F. nilgerrensis* | 0.0699 | 12.554 | 1416 | 1231 |
| *F. chinensis* | *F. moupinensis* | *F. pentaphylla* | *F. nilgerrensis* | 0.0818 | 10.462 | 1129 | 958 |
| *F. chinensis* | *F. pentaphylla* | *F. corymbosa* | *F. nilgerrensis* | 0.0733 | 9.959 | 1056 | 911 |
| *F. chinensis* | *F. moupinensis* | *F. corymbosa* | *F. nilgerrensis* | 0.0749 | 9.276 | 1246 | 1073 |
| *F. corymbosa* | *F. nubicola* | *F. pentaphylla* | *F. nilgerrensis* | 0.0637 | 8.977 | 1307 | 1150 |
| *F. chinensis* | *F. nubicola* | *F. moupinensis* | *F. nilgerrensis* | 0.0578 | 8.188 | 1262 | 1124 |
| *F. moupinensis* | *F. nubicola* | *F. pentaphylla* | *F. nilgerrensis* | 0.0419 | 7.852 | 1273 | 1171 |
| *F. chinensis* | *F. corymbosa* | *F. pentaphylla* | *F. nilgerrensis* | 0.0601 | 7.446 | 1028 | 911 |
| *F. corymbosa* | *F. moupinensis* | *F. pentaphylla* | *F. nilgerrensis* | 0.0241 | 5.087 | 1153 | 1099 |

Table S3 The model performances of CAFE analysis

| *K* | Final Likelihood (-lnL) | Lambda | values were attempted | alpha |
|---|---|---|---|---|
| 2 | 198961 | 0.089553038 | 186 values were attempted (0% rejected) | 0.721958 |
| 3 | 198180 | 0.073999117 | 155 values were attempted (0% rejected) | 0.809201 |
| 4 | 197797 | 0.066043028 | 236 values were attempted (0% rejected) | 0.850079 |
| 5 | 197580 | 0.061077893 | 341 values were attempted (0% rejected) | 0.879564 |
| 6 | 197448 | 0.058223732 | 172 values were attempted (0% rejected) | 0.932012 |

Table S4 Significantly enriched Gene Ontology terms of expanded or contracted genes relating to biological process

| Species | Status | ID | Term | Gene ratio | Corrected P-Value |
|---|---|---|---|---|---|
| *F. corymbosa* | contracted | GO:0016567 | protein ubiquitination | 0.29 | 1.01E-08 |
| *F. corymbosa* | contracted | GO:0009651 | response to salt stress | 0.31 | 4.32E-08 |
| *F. corymbosa* | contracted | GO:0009737 | response to abscisic acid | 0.29 | 2.04E-07 |
| *F. corymbosa* | contracted | GO:0009611 | response to wounding | 0.35 | 9.40E-07 |
| *F. corymbosa* | contracted | GO:0006816 | calcium ion transport | 0.77 | 1.80E-06 |
| *F. corymbosa* | contracted | GO:0009753 | response to jasmonic acid | 0.21 | 2.67E-06 |
| *F. corymbosa* | contracted | GO:0009555 | pollen development | 0.33 | 2.71E-06 |
| *F. corymbosa* | contracted | GO:0006511 | ubiquitin-dependent protein catabolic process | 0.30 | 3.24E-06 |
| *F. corymbosa* | contracted | GO:0006874 | cellular calcium ion homeostasis | 0.68 | 5.33E-06 |
| *F. corymbosa* | contracted | GO:0051603 | proteolysis involved in cellular protein catabolic process | 0.60 | 9.04E-06 |
| *F. corymbosa* | contracted | GO:0046686 | response to cadmium ion | 0.29 | 1.13E-05 |
| *F. corymbosa* | contracted | GO:0009414 | response to water deprivation | 0.29 | 1.14E-05 |
| *F. corymbosa* | contracted | GO:0071215 | cellular response to abscisic acid stimulus | 0.75 | 1.42E-05 |
| *F. corymbosa* | contracted | GO:0009409 | response to cold | 0.27 | 7.26E-05 |
| *F. corymbosa* | contracted | GO:0010150 | leaf senescence | 0.39 | 7.26E-05 |
| *F. corymbosa* | contracted | GO:0009867 | jasmonic acid mediated signaling pathway | 0.49 | 8.08E-05 |
| *F. corymbosa* | contracted | GO:0009809 | lignin biosynthetic process | 0.56 | 1.19E-04 |
| *F. corymbosa* | contracted | GO:0002229 | defense response to oomycetes | 0.22 | 2.25E-04 |
| *F. corymbosa* | contracted | GO:0009826 | unidimensional cell growth | 0.35 | 2.28E-04 |
| *F. corymbosa* | contracted | GO:0009793 | embryo development ending in seed dormancy | 0.25 | 2.29E-04 |
| *F. corymbosa* | contracted | GO:0010431 | seed maturation | 0.67 | 2.29E-04 |
| *F. corymbosa* | contracted | GO:0010087 | phloem or xylem histogenesis | 0.47 | 3.72E-04 |
| *F. corymbosa* | contracted | GO:0031408 | oxylipin biosynthetic process | 0.82 | 4.18E-04 |
| *F. corymbosa* | contracted | GO:0009620 | response to fungus | 0.36 | 7.09E-04 |
| *F. corymbosa* | contracted | GO:0009791 | post-embryonic development | 0.58 | 7.09E-04 |
| *F. corymbosa* | contracted | GO:0009733 | response to auxin | 0.27 | 7.33E-04 |
| *F. corymbosa* | contracted | GO:0010089 | xylem development | 0.54 | 8.11E-04 |

| | | | | | |
|---|---|---|---|---|---|
| *F. corymbosa* | contracted | GO:0006633 | fatty acid biosynthetic process | 0.42 | 1.01E-03 |
| *F. corymbosa* | contracted | GO:0080027 | response to herbivore | 0.86 | 1.12E-03 |
| *F. corymbosa* | contracted | GO:0034620 | cellular response to unfolded protein | 0.76 | 1.19E-03 |
| *F. corymbosa* | contracted | GO:0016125 | sterol metabolic process | 0.49 | 1.49E-03 |
| *F. corymbosa* | contracted | GO:0009639 | response to red or far red light | 0.57 | 1.96E-03 |
| *F. corymbosa* | contracted | GO:0051085 | chaperone cofactor-dependent protein refolding | 0.47 | 2.30E-03 |
| *F. corymbosa* | contracted | GO:0006508 | proteolysis | 0.30 | 2.52E-03 |
| *F. corymbosa* | contracted | GO:0009695 | jasmonic acid biosynthetic process | 0.58 | 2.77E-03 |
| *F. corymbosa* | contracted | GO:0031348 | negative regulation of defense response | 0.58 | 2.77E-03 |
| *F. corymbosa* | contracted | GO:0030244 | cellulose biosynthetic process | 0.46 | 3.15E-03 |
| *F. corymbosa* | contracted | GO:0050832 | defense response to fungus | 0.44 | 3.26E-03 |
| *F. corymbosa* | contracted | GO:0009626 | plant-type hypersensitive response | 0.43 | 3.97E-03 |
| *F. corymbosa* | contracted | GO:0006986 | response to unfolded protein | 0.67 | 4.41E-03 |
| *F. corymbosa* | contracted | GO:0000209 | protein polyubiquitination | 0.35 | 5.35E-03 |
| *F. corymbosa* | contracted | GO:0071230 | cellular response to amino acid stimulus | 0.59 | 5.61E-03 |
| *F. corymbosa* | contracted | GO:0009862 | systemic acquired resistance, salicylic acid mediated signaling pathway | 0.69 | 6.34E-03 |
| *F. corymbosa* | contracted | GO:0080092 | regulation of pollen tube growth | 0.27 | 6.91E-03 |
| *F. corymbosa* | contracted | GO:0009825 | multidimensional cell growth | 0.50 | 7.37E-03 |
| *F. corymbosa* | contracted | GO:0009741 | response to brassinosteroid | 0.47 | 7.80E-03 |
| *F. corymbosa* | contracted | GO:0006813 | potassium ion transport | 0.60 | 7.87E-03 |
| *F. corymbosa* | contracted | GO:0019941 | modification-dependent protein catabolic process | 0.60 | 7.87E-03 |
| *F. corymbosa* | contracted | GO:0006486 | protein glycosylation | 0.32 | 8.09E-03 |
| *F. corymbosa* | contracted | GO:0009408 | response to heat | 0.28 | 9.68E-03 |
| *F. corymbosa* | contracted | GO:0048658 | anther wall tapetum development | 0.89 | 1.20E-02 |
| *F. corymbosa* | contracted | GO:0002221 | pattern recognition receptor signaling pathway | 0.75 | 1.26E-02 |
| *F. corymbosa* | contracted | GO:0070588 | calcium ion transmembrane transport | 0.48 | 1.29E-02 |
| *F. corymbosa* | contracted | GO:0071472 | cellular response to salt stress | 0.48 | 1.29E-02 |
| *F. corymbosa* | contracted | GO:0006096 | glycolytic process | 0.38 | 1.32E-02 |
| *F. corymbosa* | contracted | GO:0009664 | plant-type cell wall organization | 0.38 | 1.32E-02 |

| | | | | | |
|---|---|---|---|---|---|
| *F. corymbosa* | contracted | GO:1900426 | positive regulation of defense response to bacterium | 0.55 | 1.32E-02 |
| *F. corymbosa* | contracted | GO:0006886 | intracellular protein transport | 0.26 | 1.35E-02 |
| *F. corymbosa* | contracted | GO:0009851 | auxin biosynthetic process | 0.58 | 1.47E-02 |
| *F. corymbosa* | contracted | GO:0045899 | positive regulation of RNA polymerase II transcription preinitiation complex assembly | 1.00 | 1.53E-02 |
| *F. corymbosa* | contracted | GO:0010200 | response to chitin | 0.28 | 1.70E-02 |
| *F. corymbosa* | contracted | GO:0006412 | translation | 0.24 | 1.72E-02 |
| *F. corymbosa* | contracted | GO:0006970 | response to osmotic stress | 0.27 | 1.83E-02 |
| *F. corymbosa* | contracted | GO:0010053 | root epidermal cell differentiation | 0.55 | 1.83E-02 |
| *F. corymbosa* | contracted | GO:0009567 | double fertilization forming a zygote and endosperm | 0.35 | 1.84E-02 |
| *F. corymbosa* | contracted | GO:0010498 | proteasomal protein catabolic process | 0.46 | 2.15E-02 |
| *F. corymbosa* | contracted | GO:0080156 | mitochondrial mRNA modification | 0.52 | 2.30E-02 |
| *F. corymbosa* | contracted | GO:0009740 | gibberellic acid mediated signaling pathway | 0.20 | 2.39E-02 |
| *F. corymbosa* | contracted | GO:0051762 | sesquiterpene biosynthetic process | 0.56 | 2.62E-02 |
| *F. corymbosa* | contracted | GO:0010228 | vegetative to reproductive phase transition of meristem | 0.28 | 2.76E-02 |
| *F. corymbosa* | contracted | GO:0006949 | syncytium formation | 0.60 | 2.92E-02 |
| *F. corymbosa* | contracted | GO:0009636 | response to toxic substance | 1.00 | 2.92E-02 |
| *F. corymbosa* | contracted | GO:0009957 | epidermal cell fate specification | 1.00 | 2.92E-02 |
| *F. corymbosa* | contracted | GO:0034440 | lipid oxidation | 1.00 | 2.92E-02 |
| *F. corymbosa* | contracted | GO:0070863 | positive regulation of protein exit from endoplasmic reticulum | 1.00 | 2.92E-02 |
| *F. corymbosa* | contracted | GO:0000398 | mRNA splicing, via spliceosome | 0.27 | 3.01E-02 |
| *F. corymbosa* | contracted | GO:0051865 | protein autoubiquitination | 0.67 | 3.07E-02 |
| *F. corymbosa* | contracted | GO:0042631 | cellular response to water deprivation | 0.43 | 3.07E-02 |
| *F. corymbosa* | contracted | GO:0045892 | negative regulation of transcription, DNA-templated | 0.25 | 3.09E-02 |
| *F. corymbosa* | contracted | GO:0080188 | gene silencing by RNA-directed DNA methylation | 0.53 | 3.22E-02 |
| *F. corymbosa* | contracted | GO:0042742 | defense response to bacterium | 0.37 | 3.47E-02 |
| *F. corymbosa* | contracted | GO:0010182 | sugar mediated signaling pathway | 0.42 | 3.65E-02 |
| *F. corymbosa* | contracted | GO:0016126 | sterol biosynthetic process | 0.42 | 3.65E-02 |
| *F. corymbosa* | contracted | GO:0042026 | protein refolding | 0.42 | 3.65E-02 |

| | | | | | |
|---|---|---|---|---|---|
| *F. corymbosa* | contracted | GO:2000008 | regulation of protein localization to cell surface | 0.44 | 3.65E-02 |
| *F. corymbosa* | contracted | GO:0009932 | cell tip growth | 0.62 | 4.09E-02 |
| *F. corymbosa* | contracted | GO:0003400 | regulation of COPII vesicle coating | 0.86 | 4.21E-02 |
| *F. corymbosa* | contracted | GO:0006629 | lipid metabolic process | 0.30 | 4.21E-02 |
| *F. corymbosa* | contracted | GO:0043622 | cortical microtubule organization | 0.39 | 4.21E-02 |
| *F. corymbosa* | contracted | GO:0048868 | pollen tube development | 0.43 | 4.24E-02 |
| *F. corymbosa* | contracted | GO:0071456 | cellular response to hypoxia | 0.24 | 4.28E-02 |
| *F. corymbosa* | contracted | GO:0043161 | proteasome-mediated ubiquitin-dependent protein catabolic process | 0.28 | 4.36E-02 |
| *F. corymbosa* | contracted | GO:0006401 | RNA catabolic process | 0.53 | 4.47E-02 |
| *F. corymbosa* | contracted | GO:0010015 | root morphogenesis | 0.53 | 4.47E-02 |
| *F. corymbosa* | contracted | GO:0009828 | plant-type cell wall loosening | 0.38 | 4.81E-02 |
| *F. corymbosa* | contracted | GO:0009958 | positive gravitropism | 0.38 | 4.81E-02 |
| *F. corymbosa* | expanded | GO:0018105 | peptidyl-serine phosphorylation | 0.27 | 2.62E-07 |
| *F. corymbosa* | expanded | GO:0046777 | protein autophosphorylation | 0.18 | 2.65E-06 |
| *F. corymbosa* | expanded | GO:0009813 | flavonoid biosynthetic process | 0.52 | 2.67E-06 |
| *F. corymbosa* | expanded | GO:0090332 | stomatal closure | 0.67 | 6.27E-06 |
| *F. corymbosa* | expanded | GO:0006468 | protein phosphorylation | 0.09 | 5.89E-05 |
| *F. corymbosa* | expanded | GO:0010193 | response to ozone | 0.37 | 1.00E-04 |
| *F. corymbosa* | expanded | GO:0030154 | cell differentiation | 0.20 | 3.22E-04 |
| *F. corymbosa* | expanded | GO:0051604 | protein maturation | 0.86 | 5.10E-04 |
| *F. corymbosa* | expanded | GO:0009751 | response to salicylic acid | 0.17 | 1.03E-03 |
| *F. corymbosa* | expanded | GO:0035556 | intracellular signal transduction | 0.13 | 1.07E-03 |
| *F. corymbosa* | expanded | GO:0009723 | response to ethylene | 0.17 | 1.23E-03 |
| *F. corymbosa* | expanded | GO:0010072 | primary shoot apical meristem specification | 0.47 | 1.43E-03 |
| *F. corymbosa* | expanded | GO:0009629 | response to gravity | 0.83 | 2.46E-03 |
| *F. corymbosa* | expanded | GO:0006635 | fatty acid beta-oxidation | 0.28 | 2.62E-03 |
| *F. corymbosa* | expanded | GO:0009734 | auxin-activated signaling pathway | 0.24 | 3.34E-03 |
| *F. corymbosa* | expanded | GO:0010090 | trichome morphogenesis | 0.26 | 3.56E-03 |
| *F. corymbosa* | expanded | GO:0009789 | positive regulation of abscisic acid-activated signaling pathway | 0.24 | 6.44E-03 |

| | | | | | |
|---|---|---|---|---|---|
| *F. corymbosa* | expanded | GO:0002181 | cytoplasmic translation | 0.23 | 6.91E-03 |
| *F. corymbosa* | expanded | GO:0006281 | DNA repair | 0.14 | 6.91E-03 |
| *F. corymbosa* | expanded | GO:0055072 | iron ion homeostasis | 0.29 | 9.54E-03 |
| *F. corymbosa* | expanded | GO:0071805 | potassium ion transmembrane transport | 0.24 | 1.03E-02 |
| *F. corymbosa* | expanded | GO:0048278 | vesicle docking | 0.28 | 1.10E-02 |
| *F. corymbosa* | expanded | GO:0006559 | L-phenylalanine catabolic process | 0.45 | 1.19E-02 |
| *F. corymbosa* | expanded | GO:0031540 | regulation of anthocyanin biosynthetic process | 0.45 | 1.19E-02 |
| *F. corymbosa* | expanded | GO:0010224 | response to UV-B | 0.17 | 1.22E-02 |
| *F. corymbosa* | expanded | GO:0055114 | oxidation-reduction process | 0.09 | 1.22E-02 |
| *F. corymbosa* | expanded | GO:0006612 | protein targeting to membrane | 0.26 | 1.42E-02 |
| *F. corymbosa* | expanded | GO:0006906 | vesicle fusion | 0.26 | 1.42E-02 |
| *F. corymbosa* | expanded | GO:0009561 | megagametogenesis | 0.26 | 1.42E-02 |
| *F. corymbosa* | expanded | GO:0010628 | positive regulation of gene expression | 0.32 | 1.42E-02 |
| *F. corymbosa* | expanded | GO:0010731 | protein glutathionylation | 0.67 | 1.42E-02 |
| *F. corymbosa* | expanded | GO:0045893 | positive regulation of transcription, DNA-templated | 0.10 | 1.62E-02 |
| *F. corymbosa* | expanded | GO:0010305 | leaf vascular tissue pattern formation | 0.24 | 1.75E-02 |
| *F. corymbosa* | expanded | GO:0033384 | geranyl diphosphate biosynthetic process | 0.57 | 1.75E-02 |
| *F. corymbosa* | expanded | GO:0045337 | farnesyl diphosphate biosynthetic process | 0.57 | 1.75E-02 |
| *F. corymbosa* | expanded | GO:0045903 | positive regulation of translational fidelity | 0.57 | 1.75E-02 |
| *F. corymbosa* | expanded | GO:0061025 | membrane fusion | 0.24 | 1.75E-02 |
| *F. corymbosa* | expanded | GO:0009738 | abscisic acid-activated signaling pathway | 0.14 | 2.11E-02 |
| *F. corymbosa* | expanded | GO:0010118 | stomatal movement | 0.23 | 2.31E-02 |
| *F. corymbosa* | expanded | GO:0006605 | protein targeting | 0.50 | 2.39E-02 |
| *F. corymbosa* | expanded | GO:0006364 | rRNA processing | 0.15 | 2.52E-02 |
| *F. corymbosa* | expanded | GO:0010026 | trichome differentiation | 0.26 | 2.52E-02 |
| *F. corymbosa* | expanded | GO:0034968 | histone lysine methylation | 0.33 | 2.52E-02 |
| *F. corymbosa* | expanded | GO:0007031 | peroxisome organization | 0.25 | 2.94E-02 |
| *F. corymbosa* | expanded | GO:0010167 | response to nitrate | 0.31 | 3.02E-02 |
| *F. corymbosa* | expanded | GO:0016558 | protein import into peroxisome matrix | 0.44 | 3.02E-02 |

| | | | | | |
|---|---|---|---|---|---|
| *F. corymbosa* | expanded | GO:0009926 | auxin polar transport | 0.18 | 3.20E-02 |
| *F. corymbosa* | expanded | GO:0006749 | glutathione metabolic process | 0.20 | 3.55E-02 |
| *F. corymbosa* | expanded | GO:0030148 | sphingolipid biosynthetic process | 0.28 | 4.12E-02 |
| *F. corymbosa* | expanded | GO:0006268 | DNA unwinding involved in DNA replication | 0.26 | 4.78E-02 |
| *F. corymbosa* | expanded | GO:0006882 | cellular zinc ion homeostasis | 0.60 | 4.78E-02 |
| *F. corymbosa* | expanded | GO:0009660 | amyloplast organization | 0.60 | 4.78E-02 |
| *F. corymbosa* | expanded | GO:0009908 | flower development | 0.12 | 4.78E-02 |
| *F. corymbosa* | expanded | GO:0017004 | cytochrome complex assembly | 0.60 | 4.78E-02 |
| *F. corymbosa* | expanded | GO:0030322 | stabilization of membrane potential | 0.60 | 4.78E-02 |
| *F. corymbosa* | expanded | GO:0048359 | mucilage metabolic process involved in seed coat development | 0.60 | 4.78E-02 |
| *F. corymbosa* | expanded | GO:0051571 | positive regulation of histone H3-K4 methylation | 0.60 | 4.78E-02 |
| *F. corymbosa* | expanded | GO:0071786 | endoplasmic reticulum tubular network organization | 0.60 | 4.78E-02 |
| *F. corymbosa* | expanded | GO:0080024 | indolebutyric acid metabolic process | 0.60 | 4.78E-02 |
| *F. corymbosa* | expanded | GO:2000028 | regulation of photoperiodism, flowering | 0.21 | 4.78E-02 |
| *F. corymbosa* | expanded | GO:0009965 | leaf morphogenesis | 0.15 | 4.88E-02 |
| *F. moupinensis* | contracted | GO:0009737 | response to abscisic acid | 0.27 | 6.92E-08 |
| *F. moupinensis* | contracted | GO:0031348 | negative regulation of defense response | 0.85 | 3.21E-07 |
| *F. moupinensis* | contracted | GO:0009651 | response to salt stress | 0.27 | 3.67E-07 |
| *F. moupinensis* | contracted | GO:0046686 | response to cadmium ion | 0.28 | 4.99E-07 |
| *F. moupinensis* | contracted | GO:0006468 | protein phosphorylation | 0.21 | 5.06E-06 |
| *F. moupinensis* | contracted | GO:0031408 | oxylipin biosynthetic process | 0.94 | 1.11E-05 |
| *F. moupinensis* | contracted | GO:0009615 | response to virus | 0.56 | 1.89E-05 |
| *F. moupinensis* | contracted | GO:0016567 | protein ubiquitination | 0.23 | 1.92E-05 |
| *F. moupinensis* | contracted | GO:0046777 | protein autophosphorylation | 0.30 | 4.40E-05 |
| *F. moupinensis* | contracted | GO:0006816 | calcium ion transport | 0.61 | 5.92E-05 |
| *F. moupinensis* | contracted | GO:0009695 | jasmonic acid biosynthetic process | 0.65 | 1.03E-04 |
| *F. moupinensis* | contracted | GO:0009809 | lignin biosynthetic process | 0.51 | 1.31E-04 |
| *F. moupinensis* | contracted | GO:0009867 | jasmonic acid mediated signaling pathway | 0.44 | 1.34E-04 |
| *F. moupinensis* | contracted | GO:0050832 | defense response to fungus | 0.49 | 1.34E-04 |

| | | | | | |
|---|---|---|---|---|---|
| *F. moupinensis* | contracted | GO:0006511 | ubiquitin-dependent protein catabolic process | 0.24 | 2.35E-04 |
| *F. moupinensis* | contracted | GO:0009611 | response to wounding | 0.27 | 2.82E-04 |
| *F. moupinensis* | contracted | GO:0009620 | response to fungus | 0.34 | 3.19E-04 |
| *F. moupinensis* | contracted | GO:0006952 | defense response | 0.26 | 3.53E-04 |
| *F. moupinensis* | contracted | GO:0006355 | regulation of transcription, DNA-templated | 0.18 | 3.68E-04 |
| *F. moupinensis* | contracted | GO:0009733 | response to auxin | 0.25 | 5.74E-04 |
| *F. moupinensis* | contracted | GO:0009873 | ethylene-activated signaling pathway | 0.43 | 8.56E-04 |
| *F. moupinensis* | contracted | GO:0009617 | response to bacterium | 0.31 | 1.01E-03 |
| *F. moupinensis* | contracted | GO:0009846 | pollen germination | 0.40 | 1.13E-03 |
| *F. moupinensis* | contracted | GO:0009860 | pollen tube growth | 0.30 | 1.25E-03 |
| *F. moupinensis* | contracted | GO:0042742 | defense response to bacterium | 0.44 | 1.25E-03 |
| *F. moupinensis* | contracted | GO:0009965 | leaf morphogenesis | 0.38 | 1.48E-03 |
| *F. moupinensis* | contracted | GO:0010088 | phloem development | 1.00 | 1.77E-03 |
| *F. moupinensis* | contracted | GO:0070588 | calcium ion transmembrane transport | 0.52 | 1.84E-03 |
| *F. moupinensis* | contracted | GO:0009740 | gibberellic acid mediated signaling pathway | 0.44 | 1.90E-03 |
| *F. moupinensis* | contracted | GO:0009409 | response to cold | 0.22 | 1.93E-03 |
| *F. moupinensis* | contracted | GO:0009793 | embryo development ending in seed dormancy | 0.21 | 1.93E-03 |
| *F. moupinensis* | contracted | GO:0009555 | pollen development | 0.24 | 2.05E-03 |
| *F. moupinensis* | contracted | GO:0018105 | peptidyl-serine phosphorylation | 0.33 | 2.05E-03 |
| *F. moupinensis* | contracted | GO:0009416 | response to light stimulus | 0.25 | 2.51E-03 |
| *F. moupinensis* | contracted | GO:0009791 | post-embryonic development | 0.48 | 2.86E-03 |
| *F. moupinensis* | contracted | GO:0006813 | potassium ion transport | 0.60 | 2.96E-03 |
| *F. moupinensis* | contracted | GO:0009414 | response to water deprivation | 0.22 | 3.10E-03 |
| *F. moupinensis* | contracted | GO:0048544 | recognition of pollen | 0.43 | 4.39E-03 |
| *F. moupinensis* | contracted | GO:0010187 | negative regulation of seed germination | 0.52 | 6.85E-03 |
| *F. moupinensis* | contracted | GO:0006413 | translational initiation | 0.45 | 7.06E-03 |
| *F. moupinensis* | contracted | GO:0009753 | response to jasmonic acid | 0.28 | 7.13E-03 |
| *F. moupinensis* | contracted | GO:0006561 | proline biosynthetic process | 1.00 | 8.18E-03 |
| *F. moupinensis* | contracted | GO:0010053 | root epidermal cell differentiation | 0.55 | 8.18E-03 |

| | | | | | |
|---|---|---|---|---|---|
| *F. moupinensis* | contracted | GO:0019941 | modification-dependent protein catabolic process | 0.55 | 8.18E-03 |
| *F. moupinensis* | contracted | GO:0045723 | positive regulation of fatty acid biosynthetic process | 1.00 | 8.18E-03 |
| *F. moupinensis* | contracted | GO:0042538 | hyperosmotic salinity response | 0.35 | 8.47E-03 |
| *F. moupinensis* | contracted | GO:0034620 | cellular response to unfolded protein | 0.59 | 9.66E-03 |
| *F. moupinensis* | contracted | GO:0006874 | cellular calcium ion homeostasis | 0.41 | 1.00E-02 |
| *F. moupinensis* | contracted | GO:0046688 | response to copper ion | 0.64 | 1.12E-02 |
| *F. moupinensis* | contracted | GO:0000209 | protein polyubiquitination | 0.30 | 1.22E-02 |
| *F. moupinensis* | contracted | GO:0009939 | positive regulation of gibberellic acid mediated signaling pathway | 0.88 | 1.22E-02 |
| *F. moupinensis* | contracted | GO:0010047 | fruit dehiscence | 0.73 | 1.22E-02 |
| *F. moupinensis* | contracted | GO:0016197 | endosomal transport | 0.88 | 1.22E-02 |
| *F. moupinensis* | contracted | GO:0051762 | sesquiterpene biosynthetic process | 0.56 | 1.22E-02 |
| *F. moupinensis* | contracted | GO:0007017 | microtubule-based process | 0.43 | 1.25E-02 |
| *F. moupinensis* | contracted | GO:0006970 | response to osmotic stress | 0.25 | 1.26E-02 |
| *F. moupinensis* | contracted | GO:0009408 | response to heat | 0.24 | 1.29E-02 |
| *F. moupinensis* | contracted | GO:0006893 | Golgi to plasma membrane transport | 0.67 | 1.61E-02 |
| *F. moupinensis* | contracted | GO:0042753 | positive regulation of circadian rhythm | 0.67 | 1.61E-02 |
| *F. moupinensis* | contracted | GO:0007131 | reciprocal meiotic recombination | 0.41 | 1.85E-02 |
| *F. moupinensis* | contracted | GO:0006904 | vesicle docking involved in exocytosis | 0.56 | 1.86E-02 |
| *F. moupinensis* | contracted | GO:0009739 | response to gibberellin | 0.30 | 1.91E-02 |
| *F. moupinensis* | contracted | GO:0009626 | plant-type hypersensitive response | 0.35 | 1.98E-02 |
| *F. moupinensis* | contracted | GO:0006629 | lipid metabolic process | 0.28 | 2.07E-02 |
| *F. moupinensis* | contracted | GO:0009640 | photomorphogenesis | 0.31 | 2.13E-02 |
| *F. moupinensis* | contracted | GO:1900056 | negative regulation of leaf senescence | 0.62 | 2.13E-02 |
| *F. moupinensis* | contracted | GO:0007143 | female meiotic nuclear division | 0.86 | 2.60E-02 |
| *F. moupinensis* | contracted | GO:0009741 | response to brassinosteroid | 0.38 | 2.60E-02 |
| *F. moupinensis* | contracted | GO:0002229 | defense response to oomycetes | 0.18 | 2.72E-02 |
| *F. moupinensis* | contracted | GO:0010311 | lateral root formation | 0.32 | 2.72E-02 |
| *F. moupinensis* | contracted | GO:0006368 | transcription elongation from RNA polymerase II promoter | 0.57 | 2.89E-02 |
| *F. moupinensis* | contracted | GO:0006986 | response to unfolded protein | 0.50 | 3.04E-02 |

| | | | | | |
|---|---|---|---|---|---|
| *F. moupinensis* | contracted | GO:0006048 | UDP-N-acetylglucosamine biosynthetic process | 0.75 | 3.71E-02 |
| *F. moupinensis* | contracted | GO:0006370 | 7-methylguanosine mRNA capping | 1.00 | 3.71E-02 |
| *F. moupinensis* | contracted | GO:0007186 | G protein-coupled receptor signaling pathway | 0.53 | 3.71E-02 |
| *F. moupinensis* | contracted | GO:0010942 | positive regulation of cell death | 0.75 | 3.71E-02 |
| *F. moupinensis* | contracted | GO:0035336 | long-chain fatty-acyl-CoA metabolic process | 0.75 | 3.71E-02 |
| *F. moupinensis* | contracted | GO:2000377 | regulation of reactive oxygen species metabolic process | 0.47 | 3.71E-02 |
| *F. moupinensis* | contracted | GO:0006508 | proteolysis | 0.23 | 3.74E-02 |
| *F. moupinensis* | contracted | GO:0009828 | plant-type cell wall loosening | 0.35 | 4.14E-02 |
| *F. moupinensis* | contracted | GO:0090333 | regulation of stomatal closure | 0.39 | 4.19E-02 |
| *F. moupinensis* | contracted | GO:0001709 | cell fate determination | 0.58 | 4.40E-02 |
| *F. moupinensis* | contracted | GO:0009585 | red, far-red light phototransduction | 0.45 | 4.56E-02 |
| *F. moupinensis* | expanded | GO:0051603 | proteolysis involved in cellular protein catabolic process | 0.49 | 5.84E-09 |
| *F. moupinensis* | expanded | GO:0009750 | response to fructose | 0.80 | 1.87E-06 |
| *F. moupinensis* | expanded | GO:0010150 | leaf senescence | 0.26 | 2.13E-06 |
| *F. moupinensis* | expanded | GO:0007568 | aging | 0.54 | 8.78E-06 |
| *F. moupinensis* | expanded | GO:0010224 | response to UV-B | 0.30 | 2.31E-05 |
| *F. moupinensis* | expanded | GO:0009744 | response to sucrose | 0.28 | 1.46E-04 |
| *F. moupinensis* | expanded | GO:0006351 | transcription, DNA-templated | 0.22 | 2.00E-04 |
| *F. moupinensis* | expanded | GO:0009749 | response to glucose | 0.30 | 4.22E-04 |
| *F. moupinensis* | expanded | GO:0006979 | response to oxidative stress | 0.14 | 5.46E-04 |
| *F. moupinensis* | expanded | GO:0009735 | response to cytokinin | 0.27 | 6.75E-04 |
| *F. moupinensis* | expanded | GO:0006281 | DNA repair | 0.18 | 8.20E-04 |
| *F. moupinensis* | expanded | GO:0006412 | translation | 0.14 | 1.06E-03 |
| *F. moupinensis* | expanded | GO:0009723 | response to ethylene | 0.20 | 1.09E-03 |
| *F. moupinensis* | expanded | GO:0019430 | removal of superoxide radicals | 0.64 | 1.50E-03 |
| *F. moupinensis* | expanded | GO:0035194 | post-transcriptional gene silencing by RNA | 0.86 | 1.67E-03 |
| *F. moupinensis* | expanded | GO:0006749 | glutathione metabolic process | 0.31 | 1.86E-03 |
| *F. moupinensis* | expanded | GO:0051085 | chaperone cofactor-dependent protein refolding | 0.28 | 2.21E-03 |
| *F. moupinensis* | expanded | GO:0010014 | meristem initiation | 0.60 | 5.21E-03 |

| | | | | | |
|---|---|---|---|---|---|
| *F. moupinensis* | expanded | GO:0000723 | telomere maintenance | 0.32 | 5.93E-03 |
| *F. moupinensis* | expanded | GO:0006094 | gluconeogenesis | 0.36 | 6.40E-03 |
| *F. moupinensis* | expanded | GO:0009934 | regulation of meristem structural organization | 0.36 | 6.40E-03 |
| *F. moupinensis* | expanded | GO:0006401 | RNA catabolic process | 0.41 | 8.10E-03 |
| *F. moupinensis* | expanded | GO:0009407 | toxin catabolic process | 0.24 | 8.48E-03 |
| *F. moupinensis* | expanded | GO:0009650 | UV protection | 0.71 | 8.87E-03 |
| *F. moupinensis* | expanded | GO:0016458 | gene silencing | 0.50 | 9.00E-03 |
| *F. moupinensis* | expanded | GO:0030388 | fructose 1,6-bisphosphate metabolic process | 0.50 | 9.00E-03 |
| *F. moupinensis* | expanded | GO:0032502 | developmental process | 0.26 | 1.04E-02 |
| *F. moupinensis* | expanded | GO:0000724 | double-strand break repair via homologous recombination | 0.20 | 1.11E-02 |
| *F. moupinensis* | expanded | GO:0006378 | mRNA polyadenylation | 0.37 | 1.13E-02 |
| *F. moupinensis* | expanded | GO:0019432 | triglyceride biosynthetic process | 0.37 | 1.13E-02 |
| *F. moupinensis* | expanded | GO:0042274 | ribosomal small subunit biogenesis | 0.63 | 1.13E-02 |
| *F. moupinensis* | expanded | GO:0098656 | anion transmembrane transport | 0.46 | 1.13E-02 |
| *F. moupinensis* | expanded | GO:0006289 | nucleotide-excision repair | 0.27 | 1.15E-02 |
| *F. moupinensis* | expanded | GO:0034599 | cellular response to oxidative stress | 0.27 | 1.15E-02 |
| *F. moupinensis* | expanded | GO:0051026 | chiasma assembly | 0.43 | 1.33E-02 |
| *F. moupinensis* | expanded | GO:0009738 | abscisic acid-activated signaling pathway | 0.17 | 1.34E-02 |
| *F. moupinensis* | expanded | GO:0080156 | mitochondrial mRNA modification | 0.33 | 1.51E-02 |
| *F. moupinensis* | expanded | GO:1905392 | plant organ morphogenesis | 0.40 | 1.61E-02 |
| *F. moupinensis* | expanded | GO:0060149 | negative regulation of posttranscriptional gene silencing | 0.80 | 1.77E-02 |
| *F. moupinensis* | expanded | GO:1902065 | response to L-glutamate | 0.80 | 1.77E-02 |
| *F. moupinensis* | expanded | GO:0009699 | phenylpropanoid biosynthetic process | 0.50 | 1.83E-02 |
| *F. moupinensis* | expanded | GO:2000037 | regulation of stomatal complex patterning | 0.50 | 1.83E-02 |
| *F. moupinensis* | expanded | GO:0055114 | oxidation-reduction process | 0.10 | 1.93E-02 |
| *F. moupinensis* | expanded | GO:0046470 | phosphatidylcholine metabolic process | 0.45 | 2.35E-02 |
| *F. moupinensis* | expanded | GO:0048317 | seed morphogenesis | 0.45 | 2.35E-02 |
| *F. moupinensis* | expanded | GO:0009635 | response to herbicide | 0.67 | 2.38E-02 |
| *F. moupinensis* | expanded | GO:0071329 | cellular response to sucrose stimulus | 0.67 | 2.38E-02 |

| | | | | | |
|---|---|---|---|---|---|
| *F. moupinensis* | expanded | GO:0000460 | maturation of 5.8S rRNA | 0.42 | 2.85E-02 |
| *F. moupinensis* | expanded | GO:0030433 | ubiquitin-dependent ERAD pathway | 0.22 | 2.85E-02 |
| *F. moupinensis* | expanded | GO:0051301 | cell division | 0.16 | 3.12E-02 |
| *F. moupinensis* | expanded | GO:0048193 | Golgi vesicle transport | 0.57 | 3.20E-02 |
| *F. moupinensis* | expanded | GO:0071586 | CAAX-box protein processing | 0.57 | 3.20E-02 |
| *F. moupinensis* | expanded | GO:0010228 | vegetative to reproductive phase transition of meristem | 0.15 | 3.46E-02 |
| *F. moupinensis* | expanded | GO:0006352 | DNA-templated transcription, initiation | 0.30 | 3.69E-02 |
| *F. moupinensis* | expanded | GO:0006086 | acetyl-CoA biosynthetic process from pyruvate | 0.50 | 4.15E-02 |
| *F. moupinensis* | expanded | GO:0071035 | nuclear polyadenylation-dependent rRNA catabolic process | 0.50 | 4.15E-02 |
| *F. moupinensis* | expanded | GO:0048366 | leaf development | 0.14 | 4.30E-02 |
| *F. moupinensis* | expanded | GO:0006334 | nucleosome assembly | 0.22 | 4.31E-02 |
| *F. moupinensis* | expanded | GO:0010119 | regulation of stomatal movement | 0.18 | 4.79E-02 |
| *F. moupinensis* | expanded | GO:0071230 | cellular response to amino acid stimulus | 0.27 | 4.79E-02 |
| *F. moupinensis* | expanded | GO:0009825 | multidimensional cell growth | 0.23 | 4.93E-02 |
| *F. moupinensis* | expanded | GO:0043161 | proteasome-mediated ubiquitin-dependent protein catabolic process | 0.15 | 4.93E-02 |

Table S5 Significantly enriched Gene Ontology terms of up-regulated or down-regulated genes relating to biological process

| Species | Status | ID | Function | Gene ratio | Corrected P-Value |
|---|---|---|---|---|---|
| *F. corymbosa* | down | GO:0010235 | guard mother cell cytokinesis | 0.60 | 1.86E-03 |
| *F. corymbosa* | down | GO:0010376 | stomatal complex formation | 0.40 | 2.19E-02 |
| *F. corymbosa* | down | GO:0061087 | positive regulation of histone H3-K27 methylation | 0.40 | 2.19E-02 |
| *F. corymbosa* | down | GO:0090436 | leaf pavement cell development | 0.40 | 2.19E-02 |
| *F. corymbosa* | down | GO:0010483 | pollen tube reception | 0.40 | 2.19E-02 |
| *F. corymbosa* | down | GO:1902806 | regulation of cell cycle G1/S phase transition | 0.40 | 2.19E-02 |
| *F. corymbosa* | down | GO:0009629 | response to gravity | 0.33 | 2.68E-02 |
| *F. corymbosa* | down | GO:0042776 | proton motive force-driven mitochondrial ATP synthesis | 0.29 | 2.69E-02 |
| *F. corymbosa* | down | GO:0010052 | guard cell differentiation | 0.25 | 7.29E-03 |
| *F. corymbosa* | down | GO:1901002 | positive regulation of response to salt stress | 0.25 | 3.02E-02 |
| *F. corymbosa* | down | GO:0008033 | tRNA processing | 0.23 | 7.94E-03 |
| *F. corymbosa* | down | GO:1902584 | positive regulation of response to water deprivation | 0.22 | 3.24E-02 |
| *F. corymbosa* | down | GO:0006378 | mRNA polyadenylation | 0.21 | 1.86E-03 |
| *F. corymbosa* | down | GO:2000037 | regulation of stomatal complex patterning | 0.20 | 3.46E-02 |
| *F. corymbosa* | down | GO:0010444 | guard mother cell differentiation | 0.18 | 3.87E-02 |
| *F. corymbosa* | down | GO:0009554 | megasporogenesis | 0.17 | 4.18E-02 |
| *F. corymbosa* | down | GO:0010262 | somatic embryogenesis | 0.15 | 4.49E-02 |
| *F. corymbosa* | down | GO:0031348 | negative regulation of defense response | 0.12 | 2.19E-02 |
| *F. corymbosa* | down | GO:0009791 | post-embryonic development | 0.10 | 2.69E-02 |
| *F. corymbosa* | down | GO:0009627 | systemic acquired resistance | 0.09 | 1.57E-02 |
| *F. corymbosa* | down | GO:0009926 | auxin polar transport | 0.07 | 4.18E-02 |
| *F. corymbosa* | down | GO:0006364 | rRNA processing | 0.07 | 8.67E-03 |
| *F. corymbosa* | down | GO:0019722 | calcium-mediated signaling | 0.05 | 3.19E-02 |
| *F. corymbosa* | down | GO:0009733 | response to auxin | 0.03 | 2.69E-02 |
| *F. corymbosa* | down | GO:0009793 | embryo development ending in seed dormancy | 0.02 | 2.19E-02 |
| *F. corymbosa* | up | GO:0006418 | tRNA aminoacylation for protein translation | 0.38 | 5.37E-04 |
| *F. corymbosa* | up | GO:0010072 | primary shoot apical meristem specification | 0.20 | 4.23E-02 |

| | | | | | |
|---|---|---|---|---|---|
| *F. corymbosa* | up | GO:0016132 | brassinosteroid biosynthetic process | 0.15 | 2.35E-02 |
| *F. corymbosa* | up | GO:0009791 | post-embryonic development | 0.13 | 3.55E-02 |
| *F. corymbosa* | up | GO:0007275 | multicellular organism development | 0.09 | 3.87E-02 |
| *F. moupinensis* | down | GO:0031348 | negative regulation of defense response | 0.27 | 4.36E-05 |
| *F. moupinensis* | down | GO:2000022 | regulation of jasmonic acid mediated signaling pathway | 0.19 | 1.22E-04 |
| *F. moupinensis* | down | GO:0031347 | regulation of defense response | 0.15 | 1.22E-04 |
| *F. moupinensis* | down | GO:0009617 | response to bacterium | 0.09 | 3.05E-04 |
| *F. moupinensis* | down | GO:0090333 | regulation of stomatal closure | 0.21 | 3.17E-04 |
| *F. moupinensis* | down | GO:0051707 | response to other organism | 0.20 | 1.95E-03 |
| *F. moupinensis* | down | GO:0061077 | chaperone-mediated protein folding | 0.27 | 4.33E-03 |
| *F. moupinensis* | down | GO:0071456 | cellular response to hypoxia | 0.05 | 5.46E-03 |
| *F. moupinensis* | down | GO:0006468 | protein phosphorylation | 0.03 | 5.46E-03 |
| *F. moupinensis* | down | GO:0010015 | root morphogenesis | 0.24 | 5.46E-03 |
| *F. moupinensis* | down | GO:0042742 | defense response to bacterium | 0.04 | 5.58E-03 |
| *F. moupinensis* | down | GO:0009611 | response to wounding | 0.05 | 1.41E-02 |
| *F. moupinensis* | down | GO:0009751 | response to salicylic acid | 0.07 | 1.41E-02 |
| *F. moupinensis* | down | GO:1900056 | negative regulation of leaf senescence | 0.23 | 2.50E-02 |
| *F. moupinensis* | down | GO:0002237 | response to molecule of bacterial origin | 0.13 | 2.58E-02 |
| *F. moupinensis* | down | GO:0007186 | G protein-coupled receptor signaling pathway | 0.20 | 2.98E-02 |
| *F. moupinensis* | down | GO:0009862 | systemic acquired resistance, salicylic acid mediated signaling pathway | 0.19 | 3.41E-02 |
| *F. moupinensis* | down | GO:0010200 | response to chitin | 0.05 | 3.76E-02 |
| *F. moupinensis* | down | GO:0009789 | positive regulation of abscisic acid-activated signaling pathway | 0.11 | 3.80E-02 |
| *F. moupinensis* | down | GO:0002229 | defense response to oomycetes | 0.07 | 4.31E-02 |
| *F. moupinensis* | down | GO:0006378 | mRNA polyadenylation | 0.16 | 4.64E-02 |
| *F. moupinensis* | down | GO:0048229 | gametophyte development | 0.15 | 4.88E-02 |
| *F. moupinensis* | down | GO:0009816 | defense response to bacterium, incompatible interaction | 0.09 | 4.88E-02 |
| *F. moupinensis* | up | GO:0005982 | starch metabolic process | 0.38 | 3.16E-04 |
| *F. moupinensis* | up | GO:0016458 | gene silencing | 0.42 | 9.20E-04 |
| *F. moupinensis* | up | GO:0007004 | telomere maintenance via telomerase | 0.40 | 6.46E-03 |

| | | | | | |
|---|---|---|---|---|---|
| *F. moupinensis* | up | GO:0051321 | meiotic cell cycle | 0.17 | 1.52E-02 |
| *F. moupinensis* | up | GO:0009251 | glucan catabolic process | 0.50 | 1.74E-02 |
| *F. moupinensis* | up | GO:0006268 | DNA unwinding involved in DNA replication | 0.21 | 2.31E-02 |
| *F. moupinensis* | up | GO:0010050 | vegetative phase change | 0.38 | 2.59E-02 |
| *F. moupinensis* | up | GO:0003006 | developmental process involved in reproduction | 0.38 | 2.59E-02 |
| *F. moupinensis* | up | GO:0006388 | tRNA splicing, via endonucleolytic cleavage and ligation | 0.33 | 3.23E-02 |
| *F. moupinensis* | up | GO:0006261 | DNA-dependent DNA replication | 0.17 | 3.80E-02 |
| *F. moupinensis* | up | GO:0000724 | double-strand break repair via homologous recombination | 0.09 | 3.84E-02 |
| *F. moupinensis* | up | GO:0031425 | chloroplast RNA processing | 0.27 | 4.16E-02 |
| *F. moupinensis* | up | GO:0010073 | meristem maintenance | 0.15 | 4.16E-02 |
| *F. moupinensis* | up | GO:0010082 | regulation of root meristem growth | 0.15 | 4.16E-02 |

Table S6 The size of sequencing data of five *Fragaria* plants

| *Fragaria* species | Illumina data (Gb) and coverage | PacBio data (Gb) and coverage (×) | Hi-C data (Gb) and coverage (×) | RNA sequencing (Gb) | | | |
|---|---|---|---|---|---|---|---|
| | | | | Leaf | Root | Stolon | Stem |
| *F. corymbosa* | 33.00 (139.25 ×) | 44.64 (188.36 ×) | 27.49 (116.00 ×) | 10.60 | 7.73 | 7.52 | 8.21 |
| *F. moupinensis* | 30.50 (129.13 ×) | 40.20 (170.19 ×) | 32.37 (137.04 ×) | 7.86 | 8.80 | 7.73 | 8.02 |
| *F. chinensis* | 171.00 (723.75 ×) | 39.99 (169.26 ×) | 34.92 (147.80 ×) | 8.19 | 8.90 | 8.20 | 8.29 |
| *F. pentaphylla* | 29.32 (113.11 ×) | 39.99 (154.28 ×) | 48.62 (178.57 ×) | 7.95 | 7.27 | 8.03 | 8.18 |
| *F. daltoniana* | 31.66 (115.69 ×) | 72.00 (263.09 ×) | 38.29 (141.55 ×) | 7.60 | 8.38 | 8.63 | 8.42 |

Table S7 Whole genome resequencing data used in the present study

| *Fragaria* species | Accession ID | Source | Data size (Gb) | sppIDer analysis | *D*-statistics |
|---|---|---|---|---|---|
| *F. corymbosa* | FDES192246202 | this study | 14.71 | Yes | Yes |
| *F. corymbosa* | FDES192246204 | this study | 12.99 | Yes | Yes |
| *F. corymbosa* | SCH362D | this study | 5.67 | Yes | Yes |
| *F. corymbosa* | SRR13775102 | NCBI database | 37.16 | Yes | Yes |
| *F. corymbosa* | SRR22800220 | NCBI database | 33.68 | Yes | Yes |
| *F. corymbosa* | SRR22800221 | NCBI database | 35.39 | Yes | Yes |
| *F. corymbosa* | SRR22800223 | NCBI database | 37.94 | Yes | Yes |
| *F. corymbosa* | SRR22800224 | NCBI database | 36.29 | Yes | Yes |
| *F. corymbosa* | SRR11788127 | NCBI database | 7.22 | Yes | Yes |
| *F. corymbosa* | SRR5275236 | NCBI database | 0.31 | Yes | Yes |
| *F. chinensis* | SAH272D | this study | 0.76 | Yes | Yes |
| *F. chinensis* | SAH282C | this study | 4.16 | Yes | Yes |
| *F. chinensis* | SAH762A | this study | 6.89 | Yes | Yes |
| *F. chinensis* | SAH782B | this study | 3.97 | Yes | Yes |
| *F. chinensis* | SRR22800229 | NCBI database | 21.18 | Yes | Yes |
| *F. chinensis* | SRR22800240 | NCBI database | 20.37 | Yes | Yes |
| *F. chinensis* | SRR22800251 | NCBI database | 20.78 | Yes | Yes |
| *F. chinensis* | SRR22800252 | NCBI database | 20.56 | Yes | Yes |
| *F. chinensis* | SRR11788122 | NCBI database | 7.06 | Yes | Yes |
| *F. chinensis* | SRR5275238 | NCBI database | 2.92 | Yes | Yes |
| *F. moupinensis* | FDES192246207 | this study | 14.64 | Yes | Yes |
| *F. moupinensis* | GSH454B | this study | 15.90 | Yes | Yes |
| *F. moupinensis* | GSH494B | this study | 15.72 | Yes | Yes |
| *F. moupinensis* | QHH454A | this study | 15.42 | Yes | Yes |
| *F. moupinensis* | SCH454B | this study | 17.88 | Yes | Yes |
| *F. moupinensis* | SCH574D | this study | 14.46 | Yes | Yes |
| *F. moupinensis* | SCH734D | this study | 13.15 | Yes | Yes |

| | | | | | |
|---|---|---|---|---|---|
| *F. moupinensis* | YNH634D | this study | 15.27 | Yes | Yes |
| *F. moupinensis* | YNH644C | this study | 17.08 | Yes | Yes |
| *F. moupinensis* | YNH664B | this study | 17.95 | Yes | Yes |
| *F. pentaphylla* | FDES192246205 | this study | 4.20 | No | Yes |
| *F. pentaphylla* | GSH452A | this study | 5.83 | No | Yes |
| *F. pentaphylla* | QHH492B | this study | 4.80 | No | Yes |
| *F. pentaphylla* | QHH562C | this study | 6.42 | No | Yes |
| *F. pentaphylla* | SAH402B | this study | 4.66 | No | Yes |
| *F. pentaphylla* | SCH452A | this study | 9.06 | No | Yes |
| *F. pentaphylla* | SCH552D | this study | 6.06 | No | Yes |
| *F. pentaphylla* | XZH372B | this study | 7.06 | No | Yes |
| *F. pentaphylla* | XZH652B | this study | 4.54 | No | Yes |
| *F. pentaphylla* | YNH662A | this study | 5.73 | No | Yes |
| *F. nubicola* | SRR23638788 | NCBI database | 11.93 | No | Yes |
| *F. nubicola* | SRR23638789 | NCBI database | 12.10 | No | Yes |
| *F. nubicola* | SRR23638842 | NCBI database | 15.52 | No | Yes |
| *F. nubicola* | SRR15668349 | NCBI database | 118.62 | No | Yes |
| *F. nubicola* | NDSFP02702 | this study | 3.68 | No | Yes |
| *F. nubicola* | SRR15668350 | NCBI database | 43.13 | No | Yes |
| *F. nubicola* | SRR13775101 | NCBI database | 24.55 | No | Yes |
| *F. nubicola* | SRR11833747 | NCBI database | 44.59 | No | Yes |
| *F. nilgerrensis* | YNH742A | this study | 5.78 | No | Yes |

## Supplementary Figures

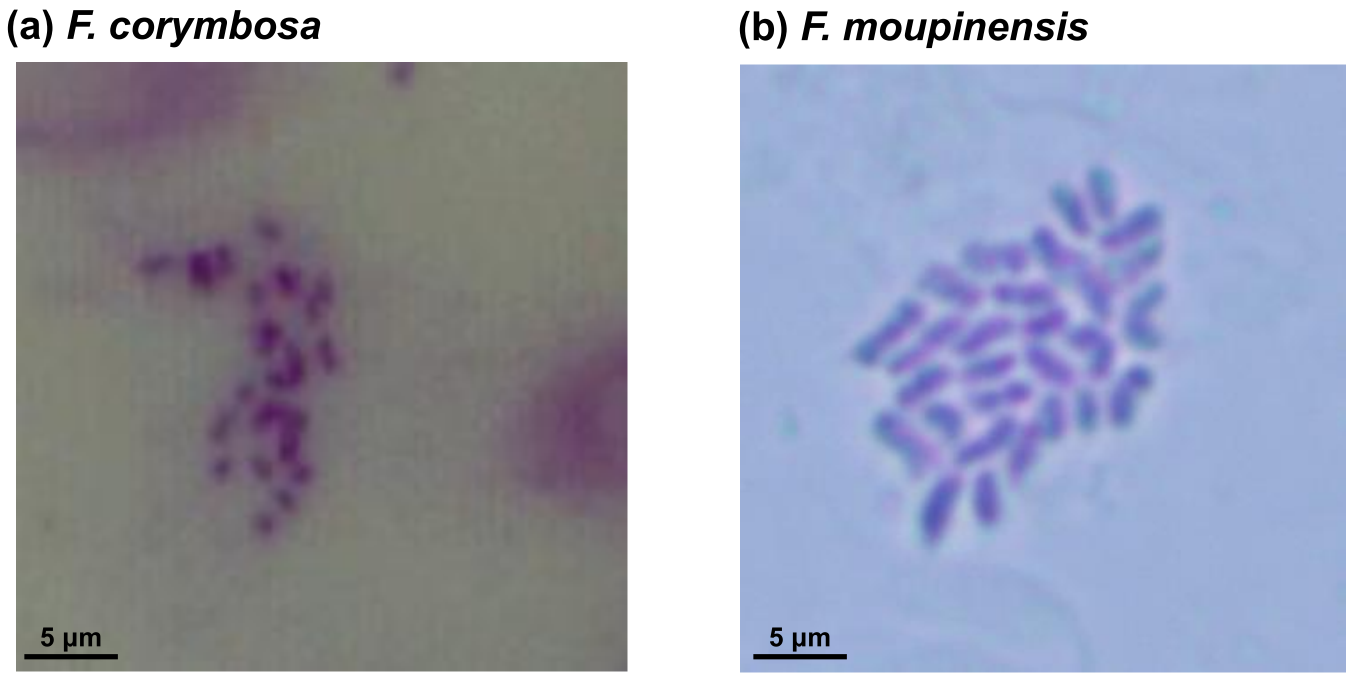


Figure S1 The micrographs of the chromosomes of (a) *F. corymbosa* and (b) *F. moupinensis* under a Motic BA310 light microscope at the 100× magnification.

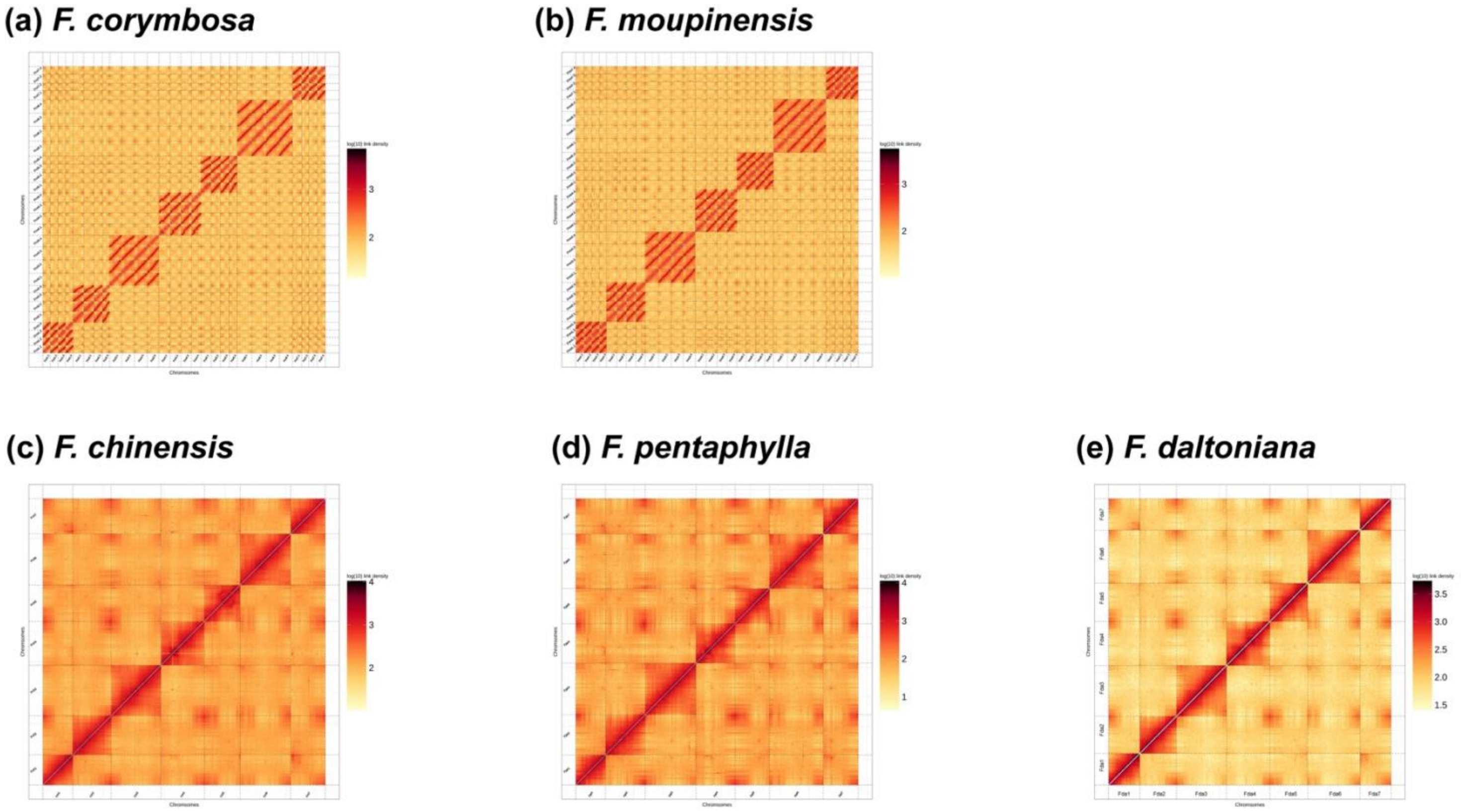


Figure S2 The Hi-C heatmaps of five sequenced *Fragaria* genomes. The scale bars indicate the density of interactions.

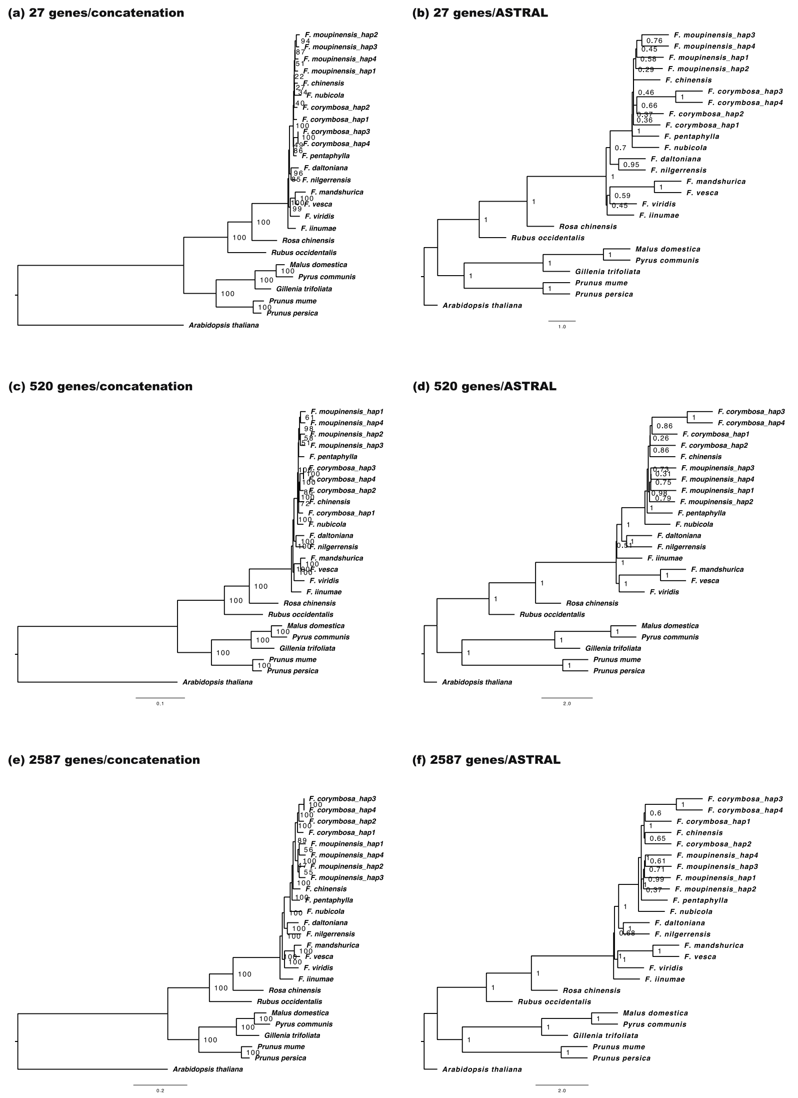


Figure S3 The reconstructed phylogeny of 19 studied species (25 haplotypes) based on both the concatenation method (ace, ML consensus trees, the left panels) and the coalescent-based method (bdf, the ASTRAL trees, the right panels) and different thresholds of haplotype coverage (ab: all haplotypes; cd: ≥ 13 haplotypes; ef: ≥ four haplotypes). In ML consensus trees (ace), the numbers represent the bootstrap values. In the ASTRAL trees (bdf), the numbers represent the local posterior probability.

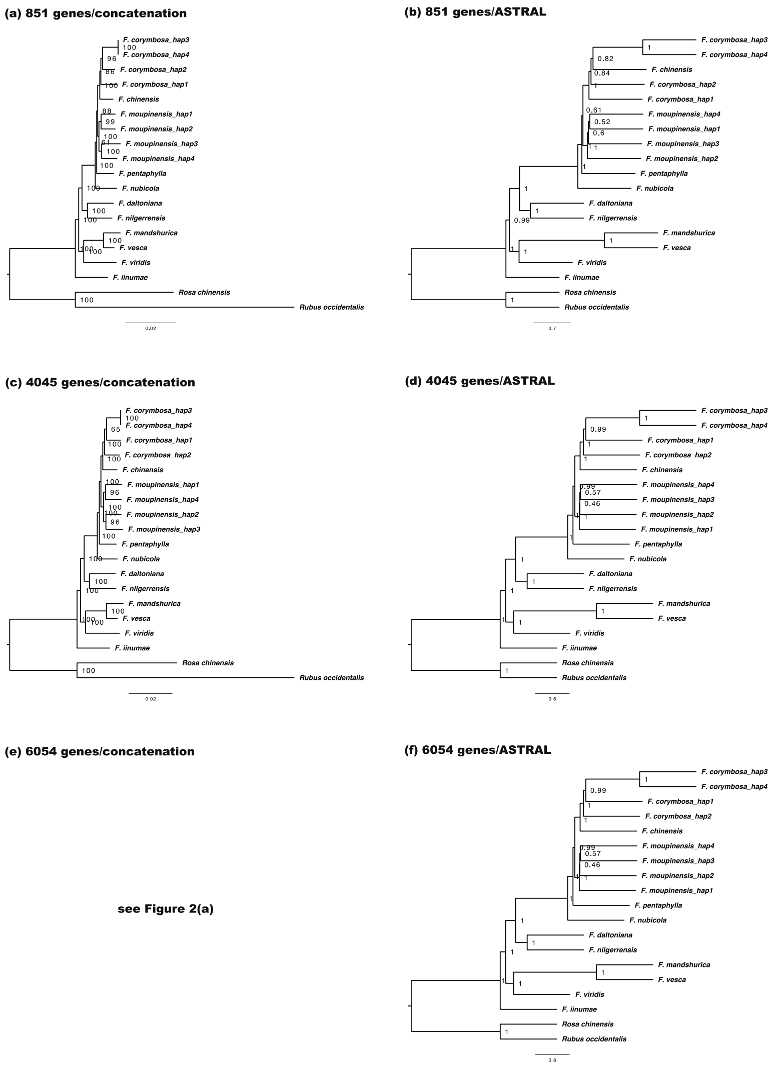


Figure S4 The reconstructed phylogeny of studied *Fragaria* and two closest species (19 haplotypes) based on both the concatenation method (ace, ML consensus trees, the left panels) and the coalescent-based method (bdf, the ASTRAL trees, the right panels) and different thresholds of haplotype coverage (ab: ≥ all haplotypes; cd: ≥ 10 haplotypes; ef: four haplotypes). In ML consensus trees (ace), the numbers represent the bootstrap values. In the ASTRAL trees (bdf), the numbers represent the local posterior probability.

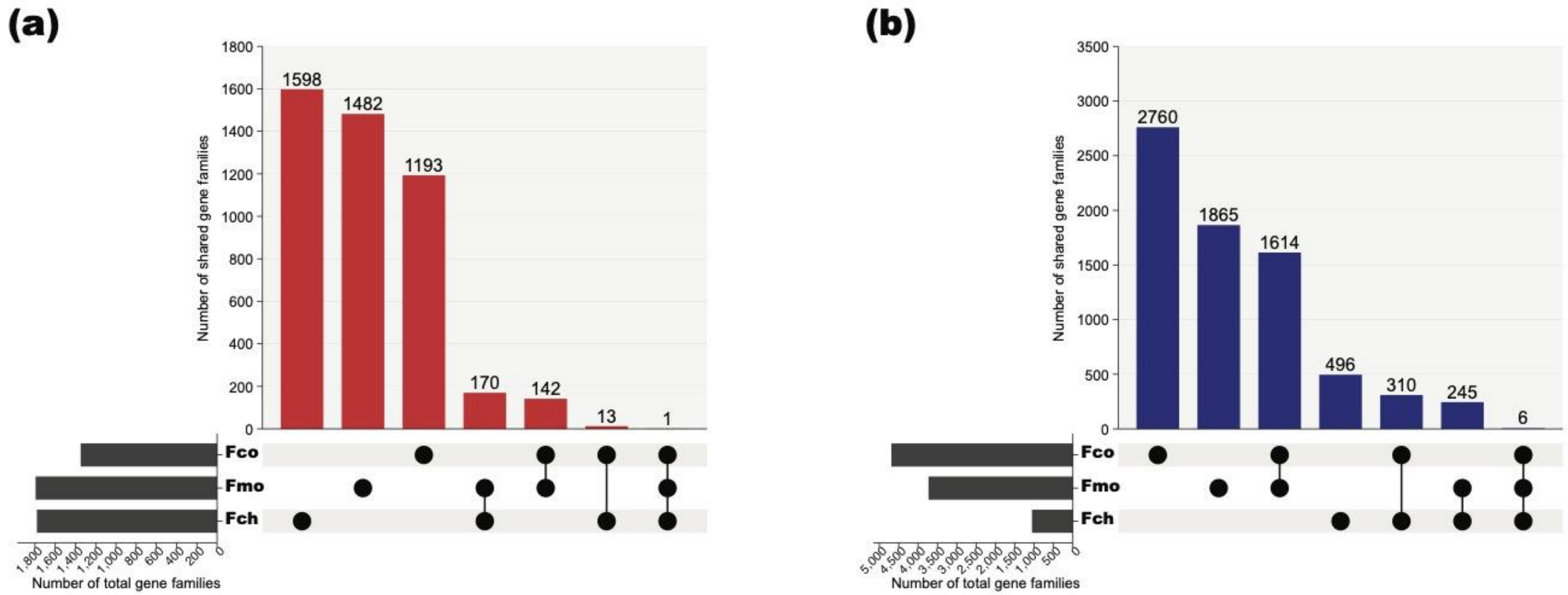


Figure S5 The number of expanded (a) and contracted (b) gene families between *Fragaria corymbosa*, *F. moupinensis*, and *F. chinensis*.

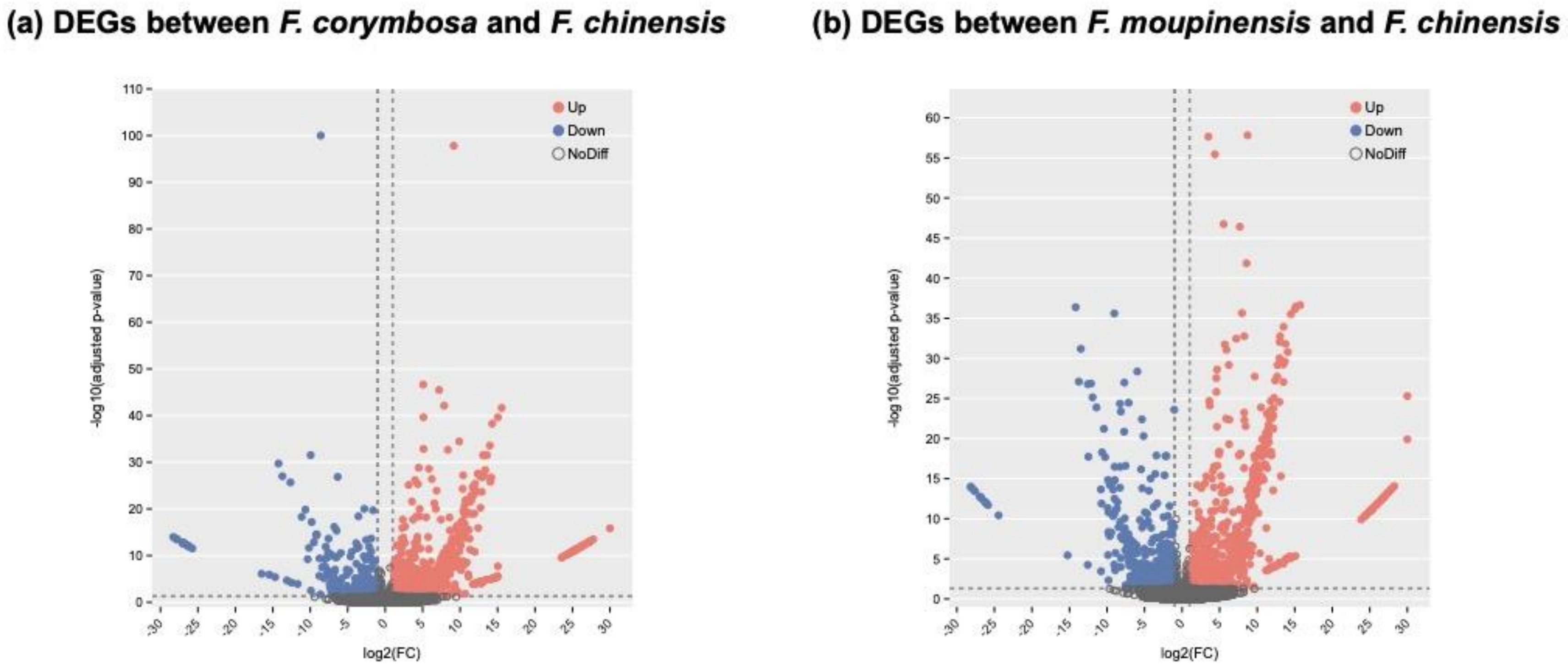


Figure S6 The volcano plots of differentially expressed genes (DEGs) between (a) *F. corymbosa* and *F. chinensis* and between (b) *F. moupinensis* and *F. chinensis*. DEGs were identified following the criteria of $|\log_2 FC| > 1$ and $p_{adj} < 0.05$. Red dots represent up-regulated genes, blue dots represent down-regulated genes, and gray dots represent genes that were not differentially expressed.
